# Effectiveness and Economic Viability of Johne's Disease (Paratuberculosis) Control Practices in Dairy Herds

**DOI:** 10.3389/fvets.2020.614727

**Published:** 2021-01-15

**Authors:** Philip Rasmussen, Herman W. Barkema, David C. Hall

**Affiliations:** ^1^Department of Ecosystem and Public Health, University of Calgary, Calgary, AB, Canada; ^2^Department of Production Animal Health, University of Calgary, Calgary, AB, Canada

**Keywords:** MAP, Johne's disease, paratuberculosis, vaccination, testing and culling, control practice, Markov chain, economic analysis

## Abstract

Johne's disease (JD or paratuberculosis) control programs have been established in many dairy-producing regions. However, the effectiveness (reduction of within-herd prevalence) and the relative economic impact as measured by, for example, the ratio of benefits to costs (BCR) across a comprehensive selection of regions and potential control practices require further investigation. Within a Markovian framework using region-specific economic variables, it was estimated that vaccination was the most promising type of JD control practice modeled, with dual-effect vaccines (reducing shedding and providing protective immunity) having BCRs between 1.48 and 2.13 in Canada, with a break-even period of between 6.17 and 7.61 years. Dual-effect vaccines were also estimated to yield BCRs greater than one in almost all major dairy-producing regions, with greater ratios in regions characterized by above-average farm-gate prices and annual production per cow. Testing and culling was comparably effective to a dual-effect vaccine at test sensitivities >70% but would remain economically unviable in almost all regions modeled.

## Introduction

Johne's disease (JD), or paratuberculosis, is an infectious chronic inflammatory disorder of the intestines that can affect domestic and wild ruminants including dairy cattle (1). The disease is caused by an infection with *Mycobacterium avium* subspecies *paratuberculosis* (MAP), a relatively resistant bacterium (2–4). As the infection progresses in cattle, the clinical effects worsen in severity from diarrhea and reduced milk production to lethargy, hypoproteinemia, and severe emaciation (5). These clinical effects result in substantial economic losses for dairy producers (6), with decreased milk production (7, 8), decreased slaughter value (9–11), and premature culling (12, 13) among the primary sources of losses. Annual losses per cow among MAP-infected herds in the United States have been estimated at US$21 (12), US$35 (14), and up to US$79 per cow (15), while annual losses among infected herds in Canada have been estimated at CA$49 (16) and between US$35 and US$57 per cow (17). Globally, average annual losses in major dairy-producing regions have been estimated at US$33 per cow, or ~1% of gross milk revenue (17). Although national control programs have already been established in several countries including Australia, Ireland, Japan, the Netherlands, and the United States (18), there are few estimates of the economic impact of potential control practices across major dairy-producing regions. It has been estimated that an average benefit of US$8.03 per animal per year is associated with vaccination in US dairy herds (19), and it has also been suggested through simulation that the most profitable strategy in average Danish herds is no control practice at all, with testing and culling being the most profitable in low-hygiene herds (20). Similarly, a recent stochastic simulation study found that no paratuberculosis control was the highly preferred strategy in small herds with 10% initial within-herd prevalence and frequently preferred in other herd scenarios (21). Intuitively, it may seem obvious that these economic losses warrant investment in control of the disease, but the precise mechanisms of control require further investigation; there is a need to estimate the effectiveness and economic impact of potential control practices with consideration for region-specific economic characteristics. Accordingly, this study estimates the effectiveness in terms of reducing within-herd prevalence, the economic impact in terms of the ratio of benefits to costs, and the break-even period in terms of years required for benefits to equal costs of various potential JD control practices across a comprehensive selection of dairy-producing regions within a Markovian framework.

## Materials and Methods

Within the Markovian framework established in Rasmussen et al. (17), a MAP-positive herd with no intervention was modeled over a 10-year horizon. Various control practices were then introduced to the simulated herds, ranging from a vaccine that reduced shedding among MAP-positive animals to more comprehensive control programs such as a “dual-effect” vaccine (a vaccine that both reduces shedding and also provides some protective immunity) combined with annual fecal PCR testing and culling of MAP-positive animals. The herds with JD control measures in place were then simulated over a 10-year horizon and compared to a positive herd with the same economic characteristics with no intervention to determine the changes in herd structure associated with each control practice. By incorporating economic variables into the Markovian framework, the region-specific benefits per cow, costs per cow, 10-year benefit-cost-ratios (BCRs), and break-even periods of each control practice were estimated. In all scenarios, regional adoption of the control practice was assumed, meaning that the replacement pool from which annual purchased replacements were acquired was assumed to be operating under the same conditions modeled for the herd.

### Markovian Framework

The spread of MAP-infection within a dairy herd was modeled over a 10-year horizon using a MAP-positive herd model with a separately modeled replacement pool (17). In this MAP-positive model, the animal can remain negative and continue aging, become infected and continue aging, or be culled. Once an animal is infected, it can either be culled or its stage of infection can progress, regress, or remain the same. Each stage of infection is associated with a different risk of being culled, and each stage has some non-shedding, lightly-shedding, moderately-shedding, and heavily-shedding states within it. Infection pressure on animals in the herd is determined by the number and degree of shedding animals in the herd in each period, and all other potential outcomes are functions of that infection pressure. For MAP-negative animals, the probability of being culled remains the steady-state MAP-negative value according to their age category. For MAP-positive animals, the probability of being culled depends on the stage of their infection, with the probability increasing with the severity of infection. After the initial age parameters were set, the herd and pool were modeled for 50 1-year periods stabilizing with an annual cow-culling rate of 27%, a young-stock percentage (including calves <1 year) of 48%, and for a 100-cow herd, 1.36 cows and 3.07 young-stock between 1 and 2 years of age brought in from the external replacement pool each year. These numbers are similar to those observed in Canadian dairy herds, which have an average cow-culling rate between 26 and 33% (22), an average young-stock percentage of 48% (23), and purchase an average of 1.37 cows and 3.09 young-stock between 1 and 2 years of age per 100 cows per year (24). Purchased replacements enter the herd at a MAP infection prevalence according to the region's animal-level prevalence, which is determined by the product of the region's average within-herd prevalence and average herd-level prevalence. For each economic region, a baseline MAP-positive herd is then compared to a MAP-positive herds with various JD control practices in place to estimate changes in herd structure, JD prevalence, and three sources of losses associated with JD in dairy cattle: premature culls; MAP-positive animals salvaged; and MAP-positive cows producing reduced amounts of milk. Lastly, because the current efficacies of available MAP vaccines in terms of reduced shedding and protective immunity are unknown, a range of vaccine efficacies are modeled.

### Vaccine: Shedding

In this control scenario, a vaccine that reduces shedding among MAP-infected animals is administered to the entire herd at time zero and then administered to natural replacements at birth and purchased replacements at the time of purchase. Once animals are vaccinated, two main mechanisms operate: (i) the probability of an animal transitioning from a MAP-negative state to a shedding state of MAP-infection is decreased by the percentage reduction in shedding attributable to the vaccine; and (ii) the probability of an animal transitioning from a shedding state of MAP-infection to another shedding state of MAP-infection is decreased by the percentage reduction in shedding attributable to the vaccine. In other words, shedding states of MAP-infection become less likely outcomes and non-shedding states become more likely according to the MAP shedding-reducing properties of the vaccine.

### Vaccine: Protective Immunity

In this control scenario, a vaccine that provides protective immunity from MAP infection is administered to the entire herd at time zero and then administered to natural replacements at birth and purchased replacements at the time of purchase. Once animals are vaccinated, a percentage of the MAP-negative animals are provided with protective immunity and separated into a new, immune cohort within the model according to the vaccine's efficacy (expressed as a percentage). The remainder of the MAP-negative animals continue in the original non-immune cohort along with the MAP-positive animals in the herd, which although vaccinated, cannot be provided with protective immunity. Animals within the immune cohort either continue aging or are culled according the MAP-negative steady-state probability for their age but can never become infected in their lifetimes. Animals that remain in the non-immune cohort are subject to infection pressure according to the number of infected animals in the herd and the degree to which those infected animals are shedding MAP. These non-immune animals can continue to age, be culled, become infected, or have their existing infections progress, regress, or remain the same.

### Vaccine: Dual-Effect

In this control scenario, a vaccine that both reduces shedding and provides protective immunity from MAP infection is administered to the entire herd at time zero and then administered to natural replacements at birth and purchased replacements at the time of purchase. The percentage of animals that are successfully provided with protective immunity enter the immune cohort, and because they are MAP-negative and remain so for their lifetimes, are not directly affected by the shedding-reducing effects of the vaccine. MAP-negative animals that remain in the non-immune cohort are still subject to infection pressure as previously described, while MAP-positive animals in this cohort transition from period to period according to the altered transition probabilities of the shedding-reduction vaccine model.

### Testing and Culling

In this control scenario, animals aged 1–7 years are tested annually using a combination of pooled and individual fecal PCR tests. They are first tested at time zero, and then retested after each transition period (year) along with purchased replacements aged 1–3 years, which are tested only at the individual level. For all testing periods, the probability of a pooled test containing samples from an *r* number of MAP-positive animals given the pool size *n*, or *pr*(*TP*) | *C*(*n, r*), is determined using the following equation:

(1) $$\begin{array}{r} pr\left(TP\right)\;|\;C\left(n,r\right)={\frac{n!}{r!*\left(n-r\right)!}}^{*}{\left(\frac{TP_{s}}{animals_{\left(1-7\right)}}\right)}^{r}\\ {}*{\left(1-\left(\frac{TP_{s}}{animals_{\left(1-7\right)}}\right)\right)}^{n-r} \end{array}$$

where: *TP*_*s*_ equals the number of true positive animals aged 1–7 years in a shedding state and *animals*_(1−7)_ equals the number of animals aged 1–7 years in the herd. A testing pool size of five animals is assumed, or *n* = 5. Pooled tests and individual tests are assumed to share the same sensitivities and specificities, or that *se*_*p*_ = *se*_*i*_ and *sp*_*p*_ = *sp*_*i*_.

The number of true positive pools detected *TP*_*p*_ given pooled test sensitivity *se*_*p*_ is determined using the following equation:

(2) $$\begin{array}{l} TP_{p}=\sum_{r=1}^{n}\left(pr\left(TP\right)\;|\;C\left(n,r\right)\right)*\frac{animals_{\left(1-7\right)}}{n}*se_{p} \end{array}$$

The number of false-positive pools detected *FP*_*p*_ given pooled test specificity *sp*_*p*_ is determined using the following equation:

(3) $$\begin{array}{l} FP_{p}=\left(\frac{animals_{\left(1-7\right)}}{n}-TP_{p}\right)*\frac{\left(1-sp_{p}\right)}{sp_{p}} \end{array}$$

The number of individual tests required *T* given the total number of positive pools detected, including true and false-positive pools, is determined using the following equation:

(4) $$\begin{array}{l} T=\left(TP_{p}+FP_{p}\right)*n \end{array}$$

The number of true positive individuals detected *TP*_*i*_ given individual test sensitivity *se*_*i*_ is determined using the following equation:

(5) $$\begin{array}{l} TP_{i}=T*\frac{\sum_{r=1}^{n}\left(pr\left(TP\right)\;|\;C\left(n,r\right)\right)*r}{\left(\sum_{r=1}^{n}\left(pr\left(TP\right)\;|\;C\left(n,r\right)\right)+FP_{p}\right)*n}*se_{i} \end{array}$$

Finally, the number of false-positive individuals detected *FP*_*i*_ given individual test specificity *sp*_*i*_ is determined using the following equation:

(6) $$\begin{array}{l} FP_{i}=\left(T-TP_{i}\right)*\frac{\left(1-sp_{i}\right)}{sp_{i}} \end{array}$$

where the total number of culls resulting from testing and culling equals the sum of true positive and false-positive individuals detected, or *TP*_*i*_ + *FP*_*i*_. These culls are then distributed across the herd according to the herd structure in that period, with the false-positive culls coming from among the MAP-negative animals and the true positive culls coming from among the MAP-positive animals. The culled animals are then replaced with animals from the replacement pool, which is assumed to be operating under the same test-and-cull conditions.

### Economic Analyses

Benefits per cow, costs per cow, benefit-cost ratios, and break-even periods of the various control practices were estimated using general input variables, region-specific dairy sector characteristics, and region-specific economic variables (17) (also available in Supplementary Files). The following values were assumed for control-specific economic variables: a fecal PCR direct testing cost of US$40 per test, a pooled testing labor cost of 30 min per test, an individual testing labor cost of 5 min per test, a vaccination direct cost of $US20 per dose for all vaccine types, and a vaccination labor cost of 1 min per dose. After each period, the herds with control practices in place were compared to a region-specific baseline MAP-positive herd with no intervention. The reduced economic losses in the herd with control practices relative to economic losses in the herd with no intervention were recorded as economic benefits for the various control practices. Premature culling benefits were estimated by tallying additional exits in the herd with no intervention and assigning those exits a value according to their age-at-exit and associated replacement price. The aggregated labor cost of seeking out, purchasing, and introducing a replacement to the herd was also accounted for. Salvage benefits were estimated by tallying additional MAP-positive exits and assigning them a reduced salvage value according to their stage of infection. Production benefits were estimated in two different ways: (i) for the comprehensive selection of major dairy-producing regions, production benefits were measured as the value of the additional milk produced (the product of quantity and farm-gate price) by the herd due to the reduced number of MAP-positive cows; and (ii) for Canada, due to the unique market conditions that arise due to supply management, production benefits were re-estimated as the reduction in variable costs from requiring fewer cows to maintain a fixed production level. The three sources of benefits in the model (reduced premature culling losses, reduced salvage losses, and reduced production losses) were summed and divided by the number of cows in the herd to obtain an estimate of benefits per cow for each control scenario in each region.

The direct cost per dose of the vaccine was added to the labor cost per dose (i.e., time required to administer a single dose multiplied by the aggregate wage rate) to obtain an estimated total cost per dose. At time zero, the entire herd was vaccinated, with only purchased and natural replacements being vaccinated after each transition period. As overall herd health improved in the model, the culling rate decreased and animals remained in the herd for a longer period, leading to fewer doses being required over time. Each period, the total cost of vaccination was divided by the number of cows in the herd to obtain an estimate of annual vaccination costs per cow for each control practice that included vaccination in each region. Similarly, the direct cost per fecal PCR test was added to the labor cost per test, with pooled tests requiring more labor than individual tests. Syringe and alcohol swab material costs for vaccine delivery were trivial (pennies per cow) at the herd-level and were not accounted for in the simulations. However, in the case of a national or widespread JD control campaign, these costs would likely be significant when aggregated across thousands of herds. The direct cost of replacing culled animals that tested positive was added to the labor cost per replacement, with the direct cost being dependent on the age of the replacement animal. The total costs of testing and replacing animals were summed each period and divided by the number of cows in the herd to obtain an estimate of annual testing and culling costs per cow for each control scenario that included testing and culling in each region.

Annual benefits and costs per cow were discounted over time at an assumed rate of 5% per annum, averaged over the 10-year horizon to obtain the reported benefit and cost estimates. This discount rate is consistent with small private firm investment in a family enterprise, falling between a public investment return rate of ~3% (25) and a private investment return rate of ~10% (26). Similarly, the Treasury Board of Canada selected a discount rate of 7% in its 2007 Cost-Benefit Analysis Guide but noted that it would likely be reduced in future years (27). Once discounted, these benefits and costs were summed over the 10-year horizon, then divided by the sum of the costs to obtain an estimate of the benefit-cost ratio for each control scenario in each region. The annual cumulative costs were subtracted from the annual cumulative benefits, and for scenarios and regions where this value was greater than zero within the 10-year horizon, the number of years required for the benefits to equal costs were recorded to obtain an estimate of the break-even period.

### Monte Carlo Simulations

Monte Carlo simulations of 10,000 iterations were run using Palisade's @RISK software version 8.0 (28) and used to estimate the distribution of possible outcomes of the Markov chain models and their sensitivity to various input variables. For these simulations, assumptions of an initial mean within-herd prevalence of 10% and an initial mean herd-level prevalence of 50% were used in all scenarios, both with normal distributions and standard deviations of 20% of their mean values. Also assumed were mean values of 50% for the vaccine's reduction in shedding, 50% for the vaccine's protective immunity efficacy, 50% for both pooled and individual fecal PCR testing sensitivities, and 99% for testing specificities. These variables were also simulated with normal distributions but with standard deviations of 20% of their means, except for testing specificities; these were simulated with normal distributions truncated from 95 to 100% and standard deviations of 10% of their means. All general input variables, region-specific economic variables, and control-specific economic variables were assumed to have normal distributions and standard deviations of 10% of their mean values. Although the data required to determine the true standard deviations of these variables are unavailable, the selected standard deviations capture a wide range of input values without destabilizing the simulations and their results.

## Results

### Distribution of Possible Outcomes

**Figure 1:**
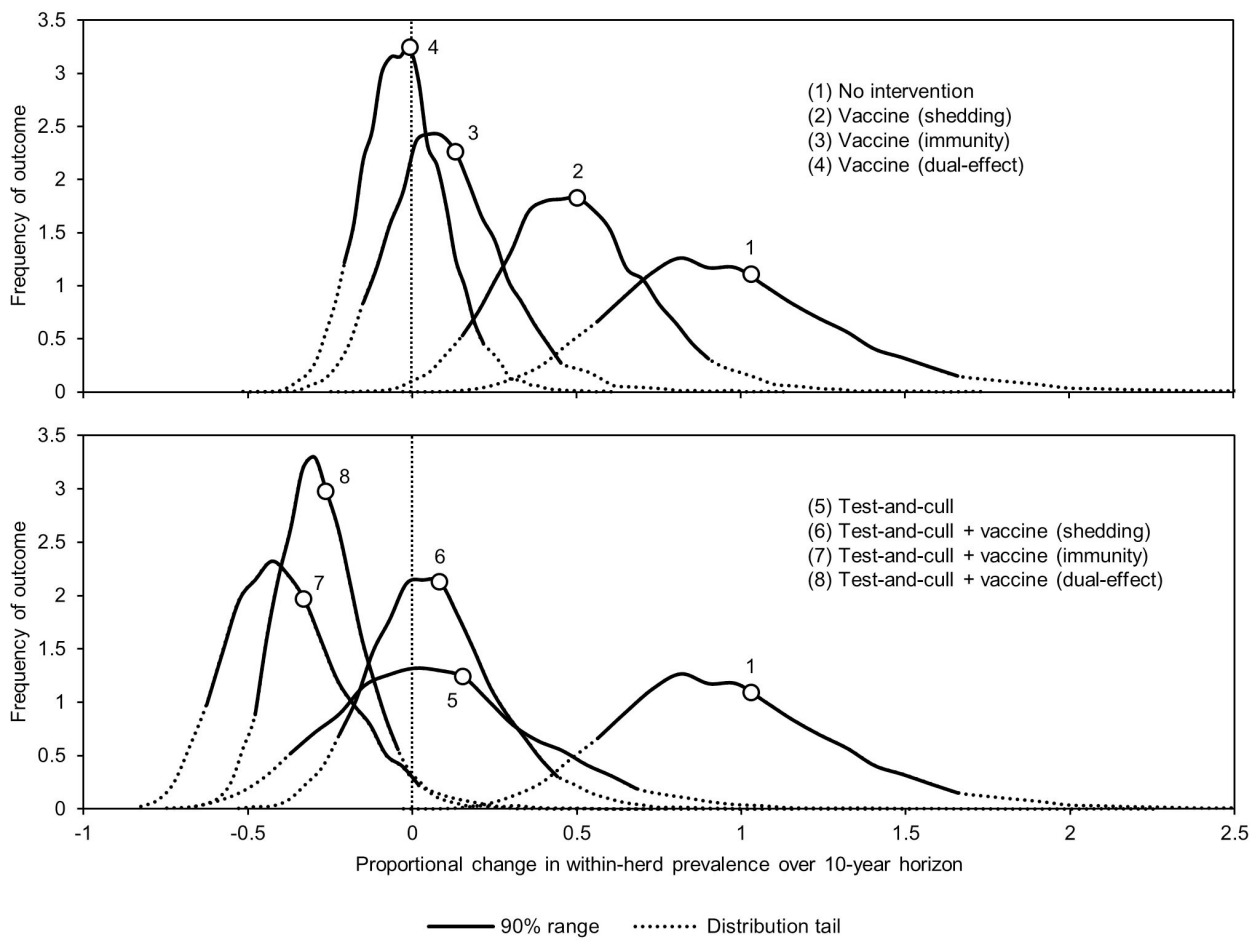
Distributions of 10-year proportional changes in within-herd prevalence for various JD (paratuberculosis) control practices compared to no intervention (10,000 iteration simulations). Assumes initial mean values of 10% for within-herd *Mycobacterium avium* subsp. *paratuberculosis* (MAP) infection prevalence, 50% for herd-level prevalence, 50% for vaccine efficacies, and 50% for testing sensitivities.

The proportional changes in within-herd prevalence (the differences between the final 10-year within-herd prevalence and the initial within-herd prevalence divided by the initial within-herd prevalence) from its initial mean value of 10% based on 10,000-iteration simulations of the various control practices are presented in Figure 1 and Table 1. For the MAP-positive herd with no intervention, 90% of the iterations resulted in proportional increases of within-herd prevalence ranging from ~0.5 to 1.65, with a mean of 1.02, equivalent to a doubling of within-herd prevalence from 10 to 20% over 10 years. Only vaccines that provided protective immunity, dual-effect vaccines, and testing and culling combined with various vaccine types had 90% confidence ranges that did not overlap with the positive herd with no intervention. Additionally, only dual-effect vaccination and testing and culling combined with either a protective immunity vaccine or a dual-effect vaccine had 90% confidence ranges entirely below zero indicative of absolute decreases in within-herd prevalence over 10 years relative to its initial value.

**Table 1:** Summary statistics of the distributions of 10-year proportional changes in within-herd *Mycobacterium avium* subsp. *paratuberculosis* (MAP) infection prevalence for various JD (paratuberculosis) control practices (10,000 iteration simulations).

**Statistic**	**No intervention**	**Vaccine (shedding)**	**Vaccine (immunity)**	**Vaccine (dual-effect)**
Minimum	−0.03	−0.25	−0.47	−0.52
Maximum	3.35	1.75	1.14	0.61
Mean	1.02	0.53	0.13	−0.13
90% range	0.51 to 1.66	0.18 to 0.92	−0.13 to 0.44	−0.22 to −0.20
Standard deviation	0.36	0.23	0.18	0.13
**Statistic**	**Test-and-cull**	**Test-and-cull with vaccine (shedding)**	**Test-and-cull with vaccine (immunity)**	**Test-and-cull with vaccine (dual-effect)**
Minimum	−0.75	−0.53	−0.83	−0.66
Maximum	2.26	1.51	1.16	0.79
Mean	0.01	0.09	−0.35	−0.26
90% range	−0.36 to 0.66	−0.20 to 0.44	−0.62 to−0.02	−0.46 to −0.04
Standard deviation	0.32	0.20	0.19	0.13

*Assumes initial mean values of 10% for within-herd MAP infection prevalence, 50% for herd-level prevalence, 50% for vaccine efficacies, and 50% for testing sensitivities*.

### Effects of JD Control on Herd Structure

**Figure 2:**
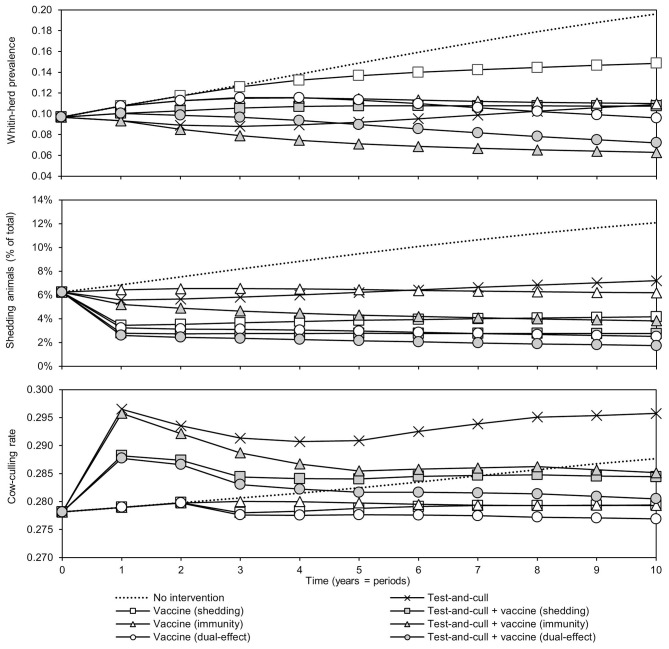
Within-herd prevalence, percentage of animals shedding, and culling rates of cows over time for various JD (paratuberculosis) control practices compared to no intervention. Assumes an initial value of 10% for within-herd *Mycobacterium avium* subsp. *paratuberculosis* (MAP) infection prevalence, 50% for herd-level prevalence, 50% for vaccine efficacies, and 50% for testing sensitivities.

The effects of the various control practices on within-herd prevalence, the percentage of shedding animals within the herd, and the cow-culling rate over time can be seen in Figure 2. In all control scenarios, prevalence decreased relative to the MAP-positive herd with no intervention. The greatest decreases relative to no intervention were observed in the scenarios of dual-effect vaccination, testing and culling combined with protective immunity vaccination, and testing and culling combined with dual-effect vaccination. After year three, the within-herd prevalence in the testing and culling scenario began to increase relative to its minimum value within the 10-year horizon. When looking at the percentage of animals shedding in the herd, overall trends are similar to those observed when looking at within-herd prevalence, including the same upward trend after year three in the testing and culling scenario. The greatest decreases were observed in the dual-effect vaccination, testing and culling combined with vaccination to reduce shedding, and testing and culling combined with dual-effect vaccination scenarios. A sharp and immediate decrease in shedding animals as a percentage of animals in the herd was observed in scenarios involving vaccines with a shedding reduction effect. As within-herd MAP prevalence and the prevalence of MAP-shedding animals changed over time in the various scenarios, so did the cow-culling rates. In the various vaccination scenarios, after 2 years the cow-culling rate began to decrease relative to the rate observed in the MAP-positive herd with no intervention, approaching the MAP-negative baseline rate of 0.275. This was indicative of both improving overall herd health and a decline in the severity of infections among MAP-positive animals as infection pressure in the herd began to fall due to the various control practices. In scenarios involving testing and culling, an initial increase in culling of cows was observed relative to the scenario with no intervention as MAP-positive animals were detected and removed from the herd. However, as the number of animals detected began to decrease with time, culling rates also fell, and by year 4, in the scenario combining testing and culling with a dual-effect vaccine, they were near or below the culling rate of cows in the positive herd with no intervention. Once again, only in the exclusive testing and culling scenario was there an eventual upward trend in the culling rate after an initial decline.

**Figure 3:**
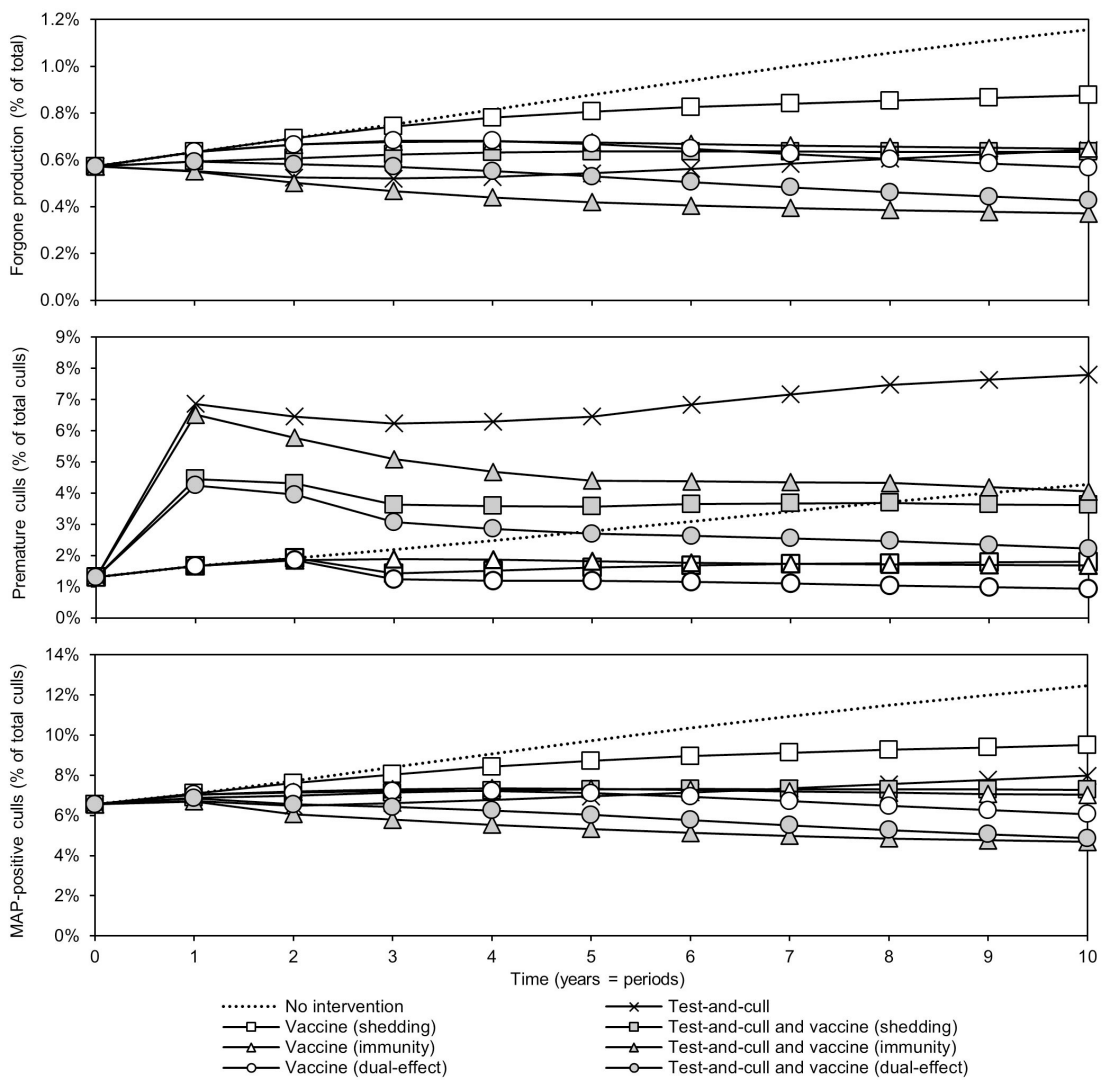
Sources of economic losses due to JD (paratuberculosis) over time for various control practices compared to no intervention. Forgone production as a percentage of potential production over time, premature culls as a percentage of total culls, and *Mycobacterium avium* subsp. *paratuberculosis* (MAP) -positive culls as a percentage of total culls. Assumes an initial value of 10% for within-herd MAP infection prevalence, 50% for herd-level prevalence, 50% for vaccine efficacies, and 50% for testing sensitivities.

Changes in the sources of economic losses in the models (forgone production, premature culling, and reduced salvage value due to MAP-positive culls) over time are presented in Figure 3. In all scenarios, forgone production, or the production lost due to MAP infection, as percentage of potential production decreased relative to the MAP-positive herd with no intervention. The greatest reductions were observed in scenarios with dual-effect vaccination and scenarios where testing and culling was combined with either a protective immunity vaccine or a dual-effect vaccine. The previously observed upward trend in the testing and culling scenario was once again observed for all sources of losses in the model. Premature culls (culls that would not have occurred in the MAP-negative baseline herd) as a percentage of total culls decreased relative to the MAP-positive herd with no intervention within 10 years in all scenarios except testing and culling, with dual-effect vaccination showing the greatest decrease. The greatest decreases in MAP-positive culls as a percentage of total culls were observed in scenarios combining testing and culling with protective immunity vaccination, testing and culling combined with dual-effect vaccination, and dual-effect vaccination only.

### Economic Analysis: Major Dairy-Producing Regions

With a 50% reduction in shedding and a 50% efficacy of protective immunity, dual-effect vaccination resulted in BCRs greater than one for all regions except Poland, Brazil, China, Russia, and Turkey with revenue-weighted average values of 1.24 and 7.88 years for the scenario's BCR and break-even period, respectively (Table 2). Even at the 90% efficacy level in the dual-effect vaccination scenario, the BCRs remain <1 for these countries. For control practices involving testing and culling (Table 3), all revenue-weighted average BCR values are less than one, with the exception of testing and culling combined with a dual-effect vaccine at the 90% efficacy and test sensitivity levels, which resulted in a BCR value of 1.22 and a break-even period of 9.17 years.

**Table 2:** Estimated benefit-cost ratios (BCRs), and revenue-weighted average benefits and costs per cow (US$), BCRs, and break-even periods (BEP) of various JD (paratuberculosis) vaccine types in major dairy-producing regions across a range of vaccine shedding reduction and protective immunity percentages.

**Region**	**Vaccine (shedding)**			**Vaccine (immunity)**			**Vaccine (dual-effect)**		
	**50%**	**70%**	**90%**	**50%**	**70%**	**90%**	**50%**	**70%**	**90%**
European Union (28)	0.69	0.94	1.18	0.99	1.32	1.60	1.23	1.47	1.60
Germany	0.80	1.10	1.37	1.14	1.52	1.84	1.43	1.70	1.85
France	0.69	0.95	1.18	0.99	1.31	1.59	1.23	1.46	1.59
Great Britain	0.73	1.00	1.26	1.07	1.42	1.72	1.32	1.57	1.71
Poland	0.36	0.51	0.65	0.62	0.82	1.01	0.73	0.87	0.95
Netherlands	0.94	1.28	1.61	1.34	1.78	2.16	1.67	1.99	2.16
Italy	0.69	0.95	1.19	1.02	1.36	1.65	1.26	1.50	1.63
Ireland	1.03	1.38	1.70	1.27	1.67	2.00	1.66	1.96	2.13
Spain	0.59	0.81	1.03	0.93	1.24	1.51	1.12	1.34	1.46
Denmark	1.06	1.45	1.80	1.49	1.98	2.40	1.87	2.22	2.42
Belgium	0.75	1.02	1.27	1.06	1.41	1.71	1.33	1.58	1.71
Austria	0.78	1.06	1.32	1.07	1.42	1.72	1.35	1.61	1.75
Czechia	0.54	0.76	0.97	0.90	1.21	1.48	1.07	1.29	1.40
Sweden	0.91	1.25	1.55	1.29	1.71	2.07	1.61	1.92	2.08
Finland	0.95	1.31	1.63	1.38	1.83	2.22	1.71	2.04	2.21
United States	0.93	1.27	1.59	1.33	1.76	2.14	1.66	1.97	2.14
California	0.91	1.24	1.56	1.31	1.73	2.10	1.63	1.93	2.10
Wisconsin	0.81	1.11	1.40	1.21	1.61	1.96	1.49	1.77	1.93
Idaho	0.70	0.97	1.23	1.12	1.50	1.83	1.35	1.61	1.75
New York	0.96	1.32	1.65	1.37	1.82	2.20	1.71	2.04	2.21
Texas	0.85	1.17	1.47	1.26	1.68	2.04	1.56	1.85	2.01
Michigan	0.79	1.10	1.39	1.24	1.65	2.02	1.50	1.80	1.95
Pennsylvania	0.79	1.08	1.35	1.14	1.51	1.84	1.42	1.69	1.83
Minnesota	0.84	1.14	1.43	1.21	1.60	1.94	1.50	1.78	1.94
New Mexico	0.75	1.04	1.32	1.18	1.57	1.92	1.43	1.71	1.86
Washington	0.91	1.25	1.57	1.33	1.76	2.14	1.65	1.96	2.13
Brazil	0.17	0.24	0.30	0.26	0.34	0.42	0.32	0.38	0.41
China	0.26	0.36	0.46	0.41	0.54	0.66	0.49	0.59	0.64
Russia	0.25	0.35	0.45	0.42	0.57	0.70	0.50	0.60	0.66
New Zealand	0.55	0.74	0.91	0.71	0.94	1.14	0.92	1.09	1.18
Turkey	0.21	0.29	0.37	0.34	0.46	0.56	0.41	0.49	0.54
Australia	0.71	0.96	1.18	0.92	1.22	1.47	1.19	1.41	1.53
Japan	1.66	2.28	2.87	2.48	3.30	4.01	3.05	3.63	3.95
**Revenue-weighted average benefits and costs (US$/cow/year), BCRs (ratio), and BEPs (years)**									
Benefit	6.20	8.48	10.60	9.00	11.94	14.47	11.14	13.24	14.37
Cost	9.05	9.02	9.01	9.05	9.03	9.02	9.02	9.00	8.99
BCR	0.69	0.94	1.18	0.99	1.32	1.60	1.24	1.47	1.60
BEP	8.38	8.67	8.22	8.47	7.60	6.89	7.88	7.05	6.58

*Assumes an initial within-herd Mycobacterium avium subsp. paratuberculosis (MAP) infection prevalence of 10% and a herd-level prevalence of 50%*.

**Table 3:** Estimated benefit-cost ratios (BCRs), and revenue-weighted average benefits and costs per cow (US$), BCRs, and break-even periods (BEP) of various JD (paratuberculosis) control practices involving testing and culling in major dairy-producing regions across a range of testing sensitivities and vaccine shedding reduction and protective immunity percentages.

**Region**	**Test-and-cull**			**Test-and-cull with vaccine (shedding)**			**Test-and-cull with vaccine (immunity)**			**Test-and-cull with vaccine (dual-effect)**		
	**50%**	**70%**	**90%**	**50%**	**70%**	**90%**	**50%**	**70%**	**90%**	**50%**	**70%**	**90%**
European Union (28)	0.44	0.54	0.59	0.42	0.52	0.58	0.59	0.71	0.79	0.69	0.85	1.07
Germany	0.46	0.56	0.60	0.47	0.57	0.65	0.64	0.74	0.82	0.76	0.93	1.19
France	0.43	0.52	0.57	0.42	0.51	0.58	0.58	0.69	0.77	0.68	0.84	1.05
Great Britain	0.46	0.57	0.62	0.45	0.55	0.62	0.63	0.75	0.84	0.73	0.90	1.13
Poland	0.39	0.53	0.62	0.30	0.37	0.38	0.48	0.64	0.78	0.49	0.63	0.73
Netherlands	0.50	0.59	0.64	0.52	0.64	0.74	0.70	0.80	0.88	0.85	1.04	1.34
Italy	0.47	0.57	0.63	0.44	0.54	0.60	0.62	0.75	0.85	0.71	0.89	1.10
Ireland	0.39	0.44	0.45	0.47	0.57	0.68	0.56	0.61	0.64	0.74	0.89	1.19
Spain	0.48	0.61	0.70	0.42	0.51	0.56	0.63	0.78	0.92	0.68	0.86	1.04
Denmark	0.51	0.60	0.64	0.56	0.68	0.79	0.73	0.83	0.89	0.90	1.11	1.44
Belgium	0.45	0.54	0.58	0.44	0.54	0.61	0.60	0.71	0.79	0.71	0.88	1.12
Austria	0.43	0.51	0.54	0.44	0.54	0.62	0.59	0.68	0.74	0.71	0.87	1.11
Czechia	0.50	0.66	0.76	0.41	0.51	0.54	0.64	0.83	0.99	0.68	0.86	1.03
Sweden	0.48	0.57	0.62	0.51	0.62	0.71	0.67	0.77	0.85	0.82	1.00	1.29
Finland	0.52	0.61	0.66	0.54	0.65	0.75	0.72	0.83	0.91	0.87	1.07	1.37
United States	0.50	0.59	0.63	0.52	0.63	0.73	0.69	0.80	0.87	0.84	1.03	1.33
California	0.50	0.59	0.64	0.51	0.63	0.72	0.69	0.80	0.88	0.83	1.03	1.32
Wisconsin	0.52	0.63	0.69	0.50	0.61	0.69	0.70	0.84	0.94	0.82	1.01	1.27
Idaho	0.55	0.69	0.78	0.48	0.60	0.65	0.72	0.89	1.03	0.80	1.00	1.23
New York	0.50	0.59	0.64	0.53	0.64	0.75	0.70	0.80	0.88	0.85	1.05	1.36
Texas	0.51	0.62	0.67	0.51	0.62	0.71	0.70	0.83	0.92	0.83	1.02	1.30
Michigan	0.55	0.68	0.76	0.52	0.64	0.71	0.75	0.90	1.03	0.85	1.06	1.32
Pennsylvania	0.47	0.57	0.62	0.47	0.57	0.65	0.64	0.76	0.84	0.76	0.94	1.19
Minnesota	0.48	0.58	0.63	0.49	0.60	0.68	0.66	0.78	0.86	0.79	0.98	1.24
New Mexico	0.54	0.67	0.75	0.50	0.61	0.68	0.72	0.88	1.01	0.82	1.02	1.26
Washington	0.51	0.61	0.66	0.52	0.64	0.73	0.71	0.82	0.91	0.85	1.05	1.34
Brazil	0.18	0.25	0.29	0.14	0.17	0.17	0.21	0.28	0.35	0.22	0.28	0.32
China	0.27	0.36	0.41	0.20	0.25	0.27	0.32	0.42	0.52	0.33	0.42	0.50
Russia	0.29	0.40	0.48	0.21	0.26	0.27	0.35	0.47	0.59	0.35	0.45	0.52
New Zealand	0.33	0.39	0.42	0.32	0.39	0.44	0.43	0.51	0.57	0.51	0.63	0.79
Turkey	0.24	0.33	0.39	0.17	0.22	0.22	0.29	0.39	0.48	0.29	0.37	0.43
Australia	0.37	0.44	0.47	0.39	0.47	0.55	0.51	0.58	0.63	0.62	0.76	0.97
Japan	0.69	0.80	0.85	0.79	0.96	1.16	1.02	1.13	1.21	1.30	1.59	2.13
**Revenue-weighted average benefits and costs (US$/cow/year), BCRs (ratio), and BEPs (years)**												
Benefit	15.36	23.82	29.45	14.29	17.25	16.34	20.84	27.16	31.10	18.26	20.60	19.46
Cost	31.65	40.59	45.98	29.85	29.41	24.65	31.52	34.79	35.68	23.49	21.41	16.01
BCR	0.49	0.59	0.64	0.48	0.59	0.66	0.66	0.78	0.87	0.78	0.96	1.22
BEP	–	–	–	–	–	10.00	10.00	10.00	10.00	10.00	10.00	9.17

*Assumes an initial within-herd Mycobacterium avium subsp. paratuberculosis (MAP) infection prevalence of 10% and a herd-level prevalence of 50%*.

### Economic Analysis: Canada

Benefits and costs for the various control practices were first estimated using the same method used for other major dairy-producing regions. They were then estimated again with consideration for the market conditions that arise due to supply management: fixed annual production and higher farm-gate prices. To account for these conditions, production losses were estimated as the increase in variable costs due to the presence of additional less productive MAP-positive cows in the herd required to maintain a fixed production level. Once again, the results are summarized using revenue-weighted average values at the bottom of each table.

With production losses measured as forgone production (Table 4), protective immunity vaccination and dual-effect vaccination scenarios resulted in mean BCRs >1 for all provinces within Canada, with the highest revenue-weighted average BCRs resulting from scenarios with dual-effect vaccination until control variables reach the 90%, when protective immunity vaccination has a slightly higher BCR. Testing and culling did not result in a BCR greater than one for any province at any test sensitivity modeled, and testing and culling combined with a shedding reduction vaccine only resulted in a BCR greater 1 in Alberta and Newfoundland and Labrador in the 90% vaccine efficacy and 90% test sensitivity scenario. Testing and culling combined with a protective immunity vaccine had a revenue weighted average BCR >1 (1.03) only at the 70% efficacy and sensitivity level, while testing and culling combined with dual-effect vaccination resulted in revenue-weighted average BCRs and provincial BCRs >1 at all vaccine efficacy and testing sensitivities modeled. Dual-effect vaccination also had the shortest break-even periods across vaccine efficacy scenarios. When production losses were instead measured as increased variable costs from additional cows in the herd being required to maintain production levels (Table 5), similar trends were observed but with lower BCRs and longer break-even periods. Dual-effect vaccination was still the most promising control practice, resulting in BCRs greater than one for all provinces with a revenue-weighted average of 1.48 in the 50% control variable scenario, and the shortest break-even periods across all efficacy and test sensitivity scenarios.

Table 4Estimated benefit-cost ratios (BCRs), and revenue-weighted average benefits and costs per cow (US$), BCRs, and break-even periods (BEP) of various JD (paratuberculosis) control practices in Canadian regions across a range of vaccine shedding reduction, protective immunity percentages, and testing sensitivities.

**Table:** 

**Region**	**Vaccine (shedding)**			**Vaccine (immunity)**			**Vaccine (dual-effect)**		
	**50%**	**70%**	**90%**	**50%**	**70%**	**90%**	**50%**	**70%**	**90%**
Canada	1.15	1.59	2.00	1.74	2.32	2.82	2.14	2.55	2.77
Québec	1.05	1.45	1.83	1.62	2.16	2.63	1.97	2.35	2.56
Ontario	1.12	1.55	1.95	1.69	2.26	2.74	2.08	2.48	2.69
British Columbia	1.26	1.74	2.19	1.92	2.55	3.11	2.34	2.80	3.04
Alberta	1.45	1.98	2.48	2.08	2.76	3.35	2.59	3.08	3.35
Manitoba	1.11	1.53	1.93	1.71	2.29	2.79	2.08	2.49	2.71
Saskatchewan	1.29	1.77	2.22	1.91	2.54	3.09	2.36	2.81	3.05
Nova Scotia	0.99	1.37	1.74	1.57	2.10	2.56	1.90	2.27	2.47
New Brunswick	0.99	1.37	1.73	1.54	2.06	2.51	1.88	2.24	2.44
Prince Edward Isl.	0.99	1.38	1.75	1.58	2.11	2.58	1.91	2.28	2.48
Nfld. and Labrador	1.56	2.15	2.70	2.34	3.12	3.80	2.88	3.43	3.73
**Revenue-weighted average benefits and costs (US$/cow/year), BCRs (ratio), and BEPs (years)**									
Benefit	10.44	14.34	18.03	15.80	21.00	25.51	19.29	22.97	24.94
Cost	9.10	9.08	9.06	9.11	9.09	9.07	9.07	9.06	9.05
BCR	1.15	1.58	1.99	1.73	2.31	2.81	2.13	2.54	2.76
BEP	9.14	7.56	6.64	7.05	5.97	5.32	6.17	5.45	5.08

**Table:** 

**Region**	**Test-and-cull**			**Test-and-cull with vaccine (shedding)**			**Test-and-cull with vaccine (immunity)**			**Test-and-cull with vaccine (dual-effect)**		
	**50%**	**70%**	**90%**	**50%**	**70%**	**90%**	**50%**	**70%**	**90%**	**50%**	**70%**	**90%**
Canada	0.61	0.73	0.79	0.64	0.79	0.91	0.87	1.00	1.10	1.05	1.30	1.68
Québec	0.62	0.75	0.82	0.62	0.76	0.87	0.86	1.02	1.13	1.02	1.27	1.61
Ontario	0.61	0.72	0.78	0.63	0.77	0.89	0.85	0.99	1.09	1.03	1.27	1.64
British Columbia	0.65	0.77	0.83	0.69	0.84	0.98	0.93	1.06	1.16	1.13	1.39	1.81
Alberta	0.60	0.69	0.74	0.69	0.84	1.01	0.88	0.98	1.04	1.13	1.37	1.84
Manitoba	0.64	0.77	0.85	0.65	0.80	0.91	0.90	1.05	1.17	1.07	1.32	1.69
Saskatchewan	0.62	0.73	0.78	0.68	0.82	0.97	0.89	1.01	1.10	1.10	1.35	1.78
Nova Scotia	0.64	0.79	0.87	0.62	0.76	0.86	0.89	1.06	1.19	1.03	1.27	1.61
New Brunswick	0.62	0.75	0.83	0.61	0.74	0.84	0.85	1.01	1.14	1.00	1.24	1.57
Prince Edward Isl.	0.65	0.79	0.88	0.62	0.77	0.86	0.89	1.06	1.20	1.03	1.28	1.61
Nfld. and Labrador	0.68	0.79	0.85	0.77	0.94	1.12	1.00	1.12	1.20	1.26	1.54	2.06
**Revenue-weighted average benefits and costs (US$/cow/year), BCRs (ratio), and BEPs (years)**												
Benefit	23.79	37.15	46.34	21.79	26.36	24.86	32.38	42.51	48.98	28.12	31.78	29.95
Cost	37.67	49.45	56.71	33.01	32.66	26.65	36.17	41.11	43.08	25.92	23.79	17.33
BCR	0.63	0.75	0.82	0.66	0.81	0.93	0.90	1.03	1.14	1.08	1.34	1.73
BEP	–	–	–	–	–	–	–	10.00	10.00	10.00	9.72	8.17

*Assumes an initial within-herd Mycobacterium avium subsp. paratuberculosis (MAP) infection prevalence of 10% and a herd-level prevalence of 50*.

Table 5Estimated benefit-cost ratios (BCRs), and revenue-weighted average benefits and costs per cow (US$), BCRs, and break-even periods (BEP) of various JD (paratuberculosis) control practices in Canadian regions across a range of vaccine shedding reduction, protective immunity percentages, and testing sensitivities, and with consideration for supply management (fixed output over time and production losses allocated as increased variable costs necessary to maintain production).

**Table:** 

**Region**	**Variable cost**^**a**^ (US$/cow/year)	**Vaccine (shedding)**			**Vaccine (immunity)**			**Vaccine (dual-effect)**		
		**50%**	**70%**	**90%**	**50%**	**70%**	**90%**	**50%**	**70%**	**90%**
Canada	2,476	0.89	1.20	1.48	1.15	1.52	1.83	1.48	1.76	1.91
Québec	2,430	0.79	1.07	1.33	1.05	1.39	1.67	1.34	1.59	1.72
Ontario	2,256	0.85	1.15	1.42	1.09	1.44	1.74	1.42	1.68	1.82
British Columbia	3,204	1.00	1.35	1.68	1.34	1.77	2.13	1.70	2.02	2.19
Alberta	3,106	1.20	1.62	1.99	1.53	2.01	2.42	1.98	2.34	2.54
Manitoba	3,014	0.87	1.18	1.46	1.18	1.57	1.89	1.50	1.78	1.93
Saskatchewan	2,785	1.02	1.37	1.69	1.31	1.73	2.08	1.69	2.00	2.17
Nova Scotia	2,515	0.73	0.99	1.23	1.00	1.32	1.59	1.26	1.50	1.63
New Brunswick	2,464	0.75	1.02	1.26	1.01	1.34	1.61	1.28	1.52	1.65
Prince Edward Isl.	2,144	0.70	0.95	1.18	0.93	1.23	1.48	1.19	1.41	1.53
Nfld. and Labrador	4,112	1.27	1.73	2.14	1.70	2.25	2.72	2.17	2.58	2.80
**Revenue-weighted average benefits and costs (US$/cow/year), BCRs (ratio), and BEPs (years)**										
	Benefit	8.04	10.85	13.38	10.48	13.80	16.59	13.44	15.88	17.21
	Cost	9.10	9.08	9.06	9.11	9.09	9.07	9.07	9.06	9.05
	BCR	0.88	1.20	1.48	1.15	1.52	1.83	1.48	1.75	1.90
	BEP	9.05	9.05	7.61	9.11	7.60	6.75	7.61	6.71	6.23

**Table:** 

**Region**	**Test-and-cull**			**Test-and-cull with accine (shedding)**			**Test-and-cull with vaccine (immunity)**			**Test-and-cull with vaccine (dual-effect)**		
	**50%**	**70%**	**90%**	**50%**	**70%**	**90%**	**50%**	**70%**	**90%**	**50%**	**70%**	**90%**
Canada	0.41	0.47	0.49	0.45	0.55	0.64	0.57	0.64	0.68	0.71	0.87	1.14
Québec	0.40	0.47	0.50	0.43	0.52	0.60	0.55	0.63	0.69	0.68	0.83	1.07
Ontario	0.39	0.45	0.47	0.44	0.53	0.62	0.55	0.61	0.65	0.69	0.84	1.09
British Columbia	0.45	0.52	0.55	0.50	0.61	0.72	0.64	0.71	0.77	0.80	0.98	1.29
Alberta	0.44	0.50	0.51	0.53	0.64	0.78	0.64	0.69	0.72	0.84	1.02	1.38
Manitoba	0.44	0.52	0.55	0.47	0.57	0.66	0.61	0.70	0.76	0.75	0.92	1.19
Saskatchewan	0.43	0.49	0.51	0.49	0.59	0.71	0.60	0.67	0.71	0.77	0.94	1.25
Nova Scotia	0.41	0.49	0.52	0.42	0.51	0.58	0.55	0.64	0.71	0.67	0.82	1.04
New Brunswick	0.40	0.48	0.51	0.42	0.51	0.59	0.55	0.64	0.70	0.67	0.82	1.05
Prince Edward Isl.	0.38	0.45	0.48	0.39	0.48	0.55	0.52	0.59	0.65	0.62	0.77	0.98
Nfld. and Labrador	0.49	0.56	0.59	0.59	0.71	0.86	0.72	0.79	0.83	0.94	1.14	1.53
**Revenue-weighted average benefits and costs (US$/cow/year), BCRs (ratio), and BEPs (years)**												
Benefit	16.23	24.68	29.79	15.73	18.86	18.08	21.83	27.86	31.36	19.59	22.00	20.91
Cost	37.92	49.83	57.17	33.15	32.79	26.74	36.37	41.38	43.39	26.02	23.89	17.38
BCR	0.43	0.50	0.52	0.47	0.58	0.68	0.60	0.67	0.72	0.75	0.92	1.20
BEP	–	–	–	–	–	–	–	–	–	–	9.26	8.48

Assumes an initial within-herd Mycobacterium avium subsp. paratuberculosis (MAP) infection prevalence of 10% and a herd-level prevalence of 50%.

a *STATCAN—Table 32-10-0136-01 Farm operating revenues and expenses, annual (29). Sum of “Feed, supplements, straw, and bedding,” “Veterinary fees, medicine, and breeding fees,” and “Salaries and wages, including benefits related to employee salaries” for average dairy farms across all revenue levels in 2018. Total per farm divided by number of cows per farm. Number of cows per farm obtained by number of cattle divided by number of farms: CDIC—Number of farms with shipments of Milk (30). Number of cattle: STATCAN—Table 32-10-0130-01—Number of cattle, by class and farm type (23)*.

### Sensitivity Analyses

**Figure 4:**
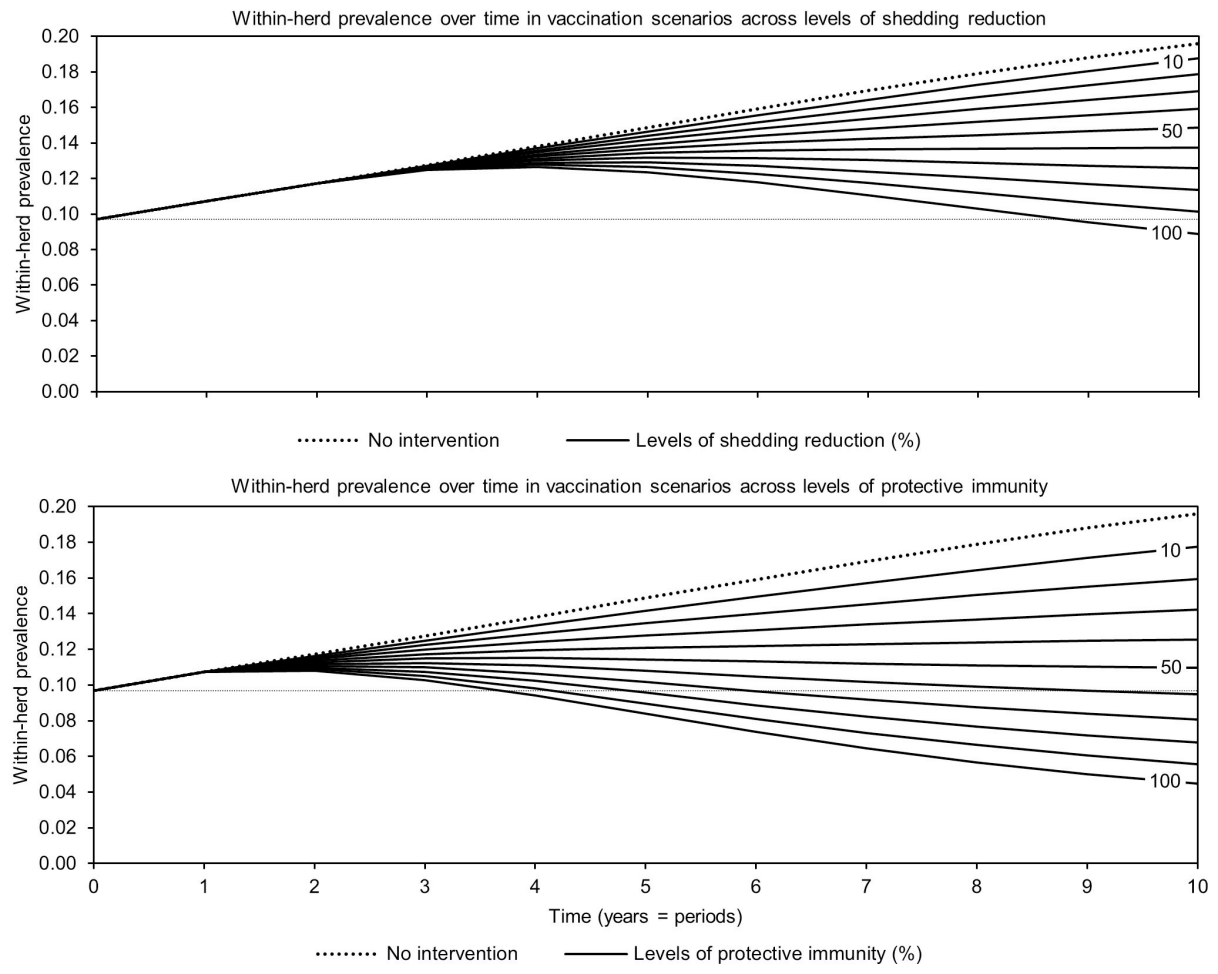
Estimates of within-herd *Mycobacterium avium* subsp. *paratuberculosis* (MAP) infection prevalence over time for JD (paratuberculosis) vaccines across a range of control-specific variable values.

**Figure 5:**
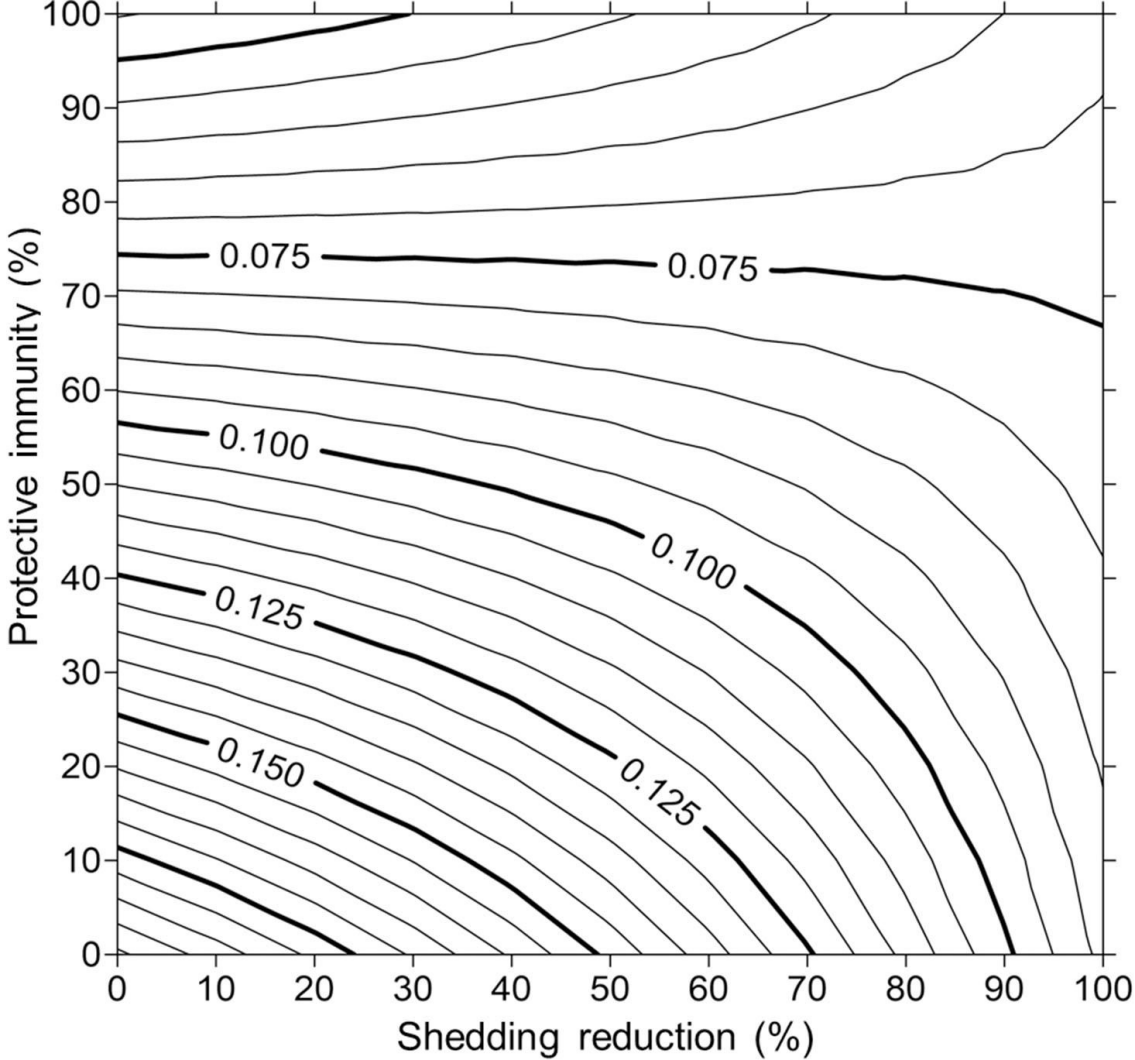
Estimates of final 10-year within-herd prevalence across a range of protective immunities and shedding reductions given an initial within-herd *Mycobacterium avium* subsp. *paratuberculosis* (MAP) infection prevalence of 0.10.

**Figure 6:**
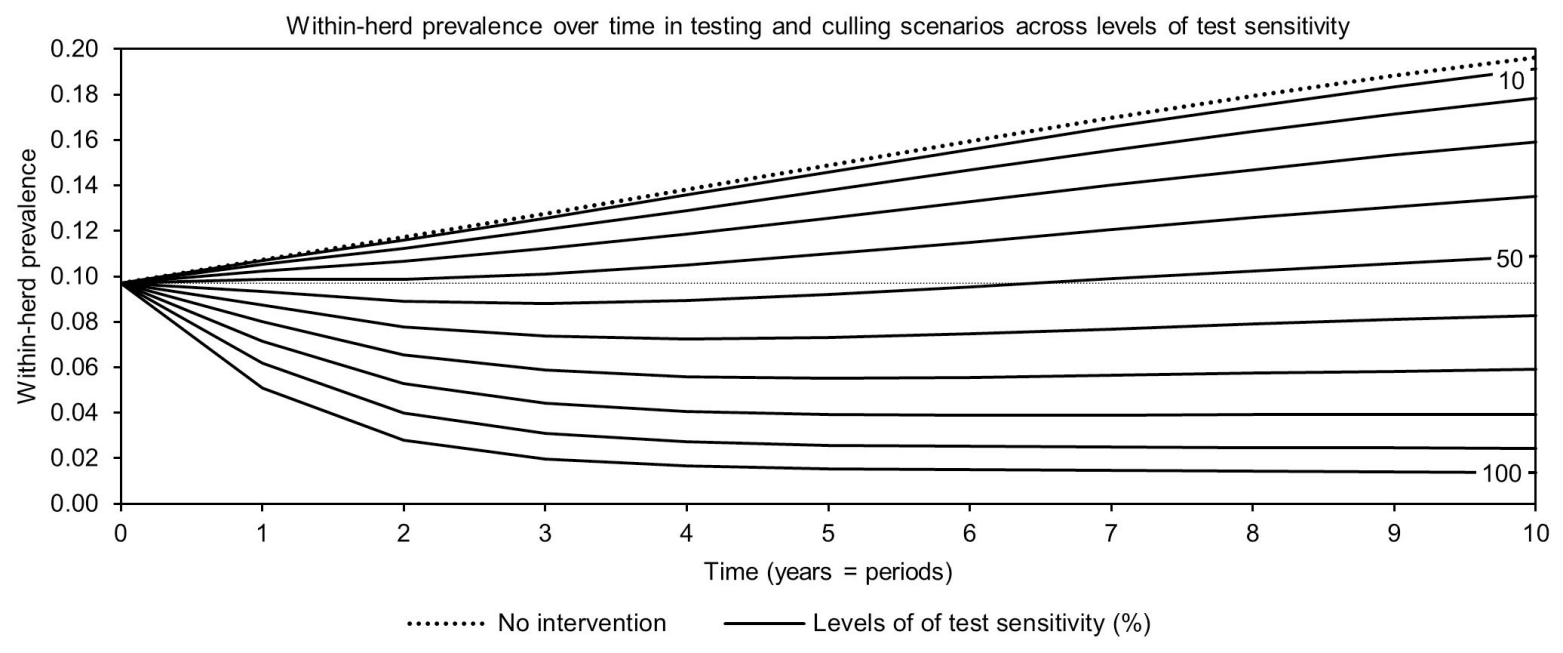
Estimates of within-herd *Mycobacterium avium* subsp. *paratuberculosis* (MAP) infection prevalence over time for testing and culling across a range of test sensitivities.

For simplicity, a generalized MAP-positive herd with no region-specific variables was selected to test the sensitivity of estimated within-herd prevalence to various input variables. For the shedding reduction vaccine, once the shedding reduction reached 70%, a slight overall downward trend in within-herd prevalence was observed (Figure 4). However, it was not until the shedding reduction exceeded 90% that an absolute decrease in within-herd prevalence relative to its initial value within the 10-year horizon was observed. For the protective immunity vaccine, at only 50% protective immunity a downward trend was observed, and an absolute decrease in within-herd prevalence within the 10-year horizon relative to its initial value was observed at <60% protective immunity. The relationship between protective immunity, shedding reduction, and the final 10-year within-herd prevalence in the dual-effect vaccination scenario is explored in Figure 5; the results suggest that the protective immunity effect drove the overall effectiveness of dual-effect vaccines in the model, particularly at moderate control variable values. For example, a vaccine with 0% shedding reduction but 70% protective immunity resulted in a final 10-year within-herd prevalence of ~0.08 (assuming an initial within-herd prevalence of 0.10), whereas a vaccine with 70% shedding reduction and 0% protective immunity resulted in a final prevalence of 0.13. There was no significant 10-year decrease in within-herd prevalence relative to its initial value resulting from testing and culling until test sensitivity exceeded 50% (Figure 6). However, even within the 50% to 70% sensitivity range, within-herd prevalence began to trend upwards in the later periods of the 10-year horizon. This upward trend did not clearly disappear until test sensitivity exceeded the 70% level.

**Figure 7:**
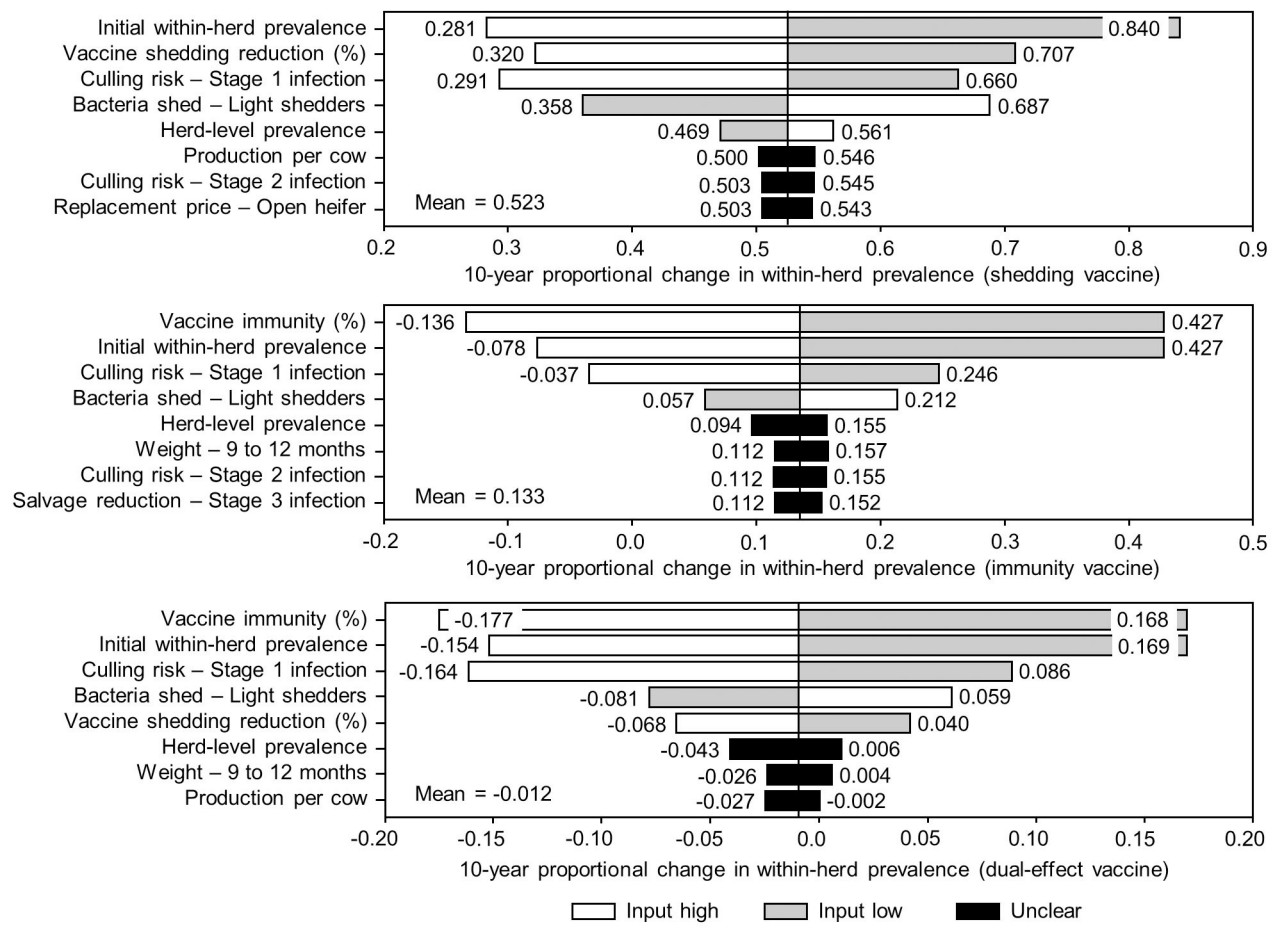
Sensitivity of 10-year proportional changes in within-herd *Mycobacterium avium* subsp. *paratuberculosis* (MAP) infection prevalence due to various JD (paratuberculosis) vaccine types to a range of input variables. Assumes initial mean values of 10% for within-herd MAP infection prevalence, 50% for herd-level prevalence, and 50% for vaccine efficacies. The color of the sensitivity bars indicates the direction of the relationship between the variable and 10-year proportional change in within-herd prevalence (grey indicates the effect of variable values below their mean value, white indicates the effect of values above their mean, and black indicates that the effect is unclear).

**Figure 8:**
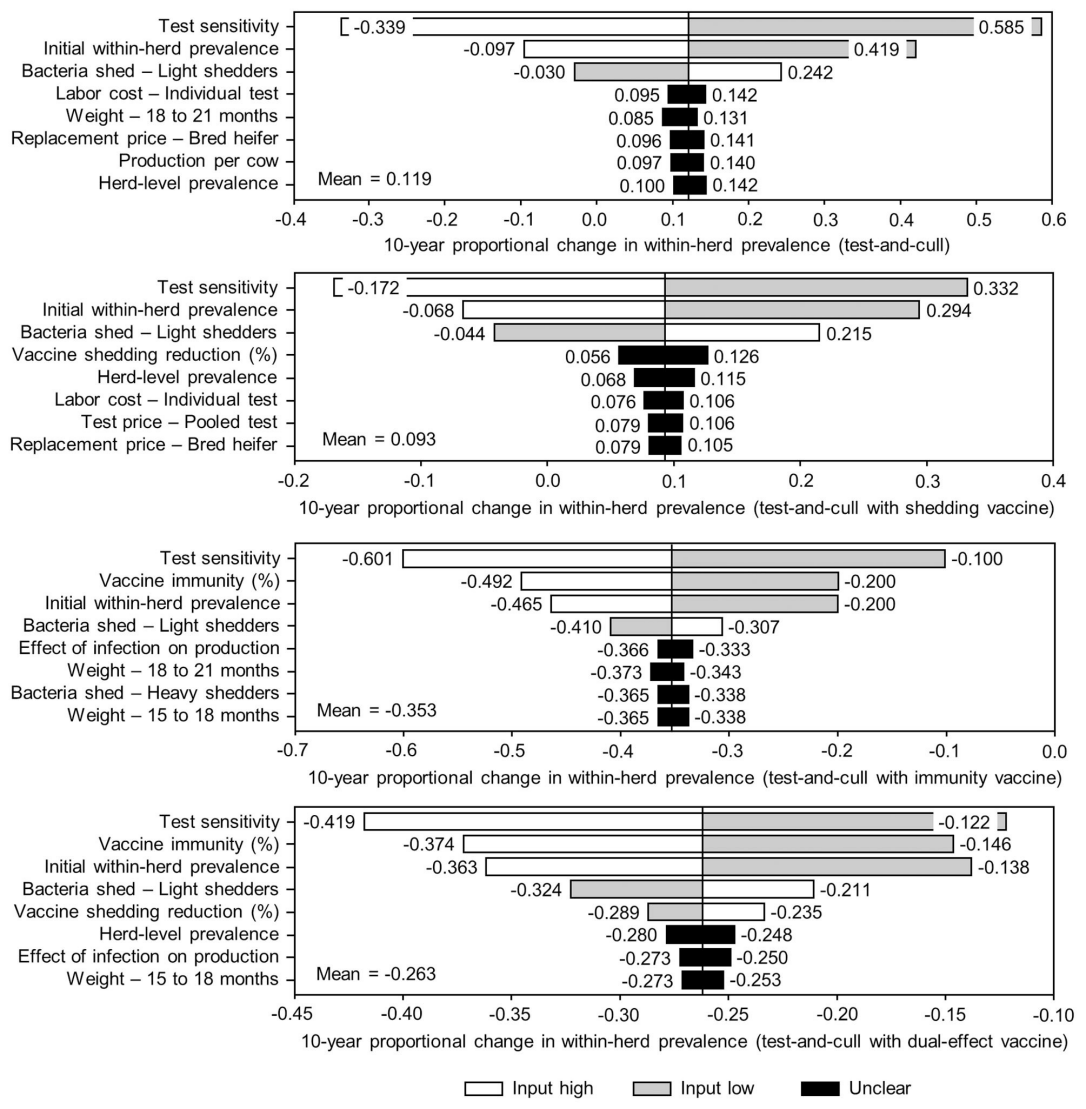
Sensitivity of 10-year proportional changes in within-herd *Mycobacterium avium* subsp. *paratuberculosis* (MAP) infection prevalence due to various JD (paratuberculosis) practices involving testing and culling to a range of input variables. Assumes initial mean values of 10% for within-herd MAP infection prevalence, 50% for herd-level prevalence, 50% for vaccine efficacies, and 50% for testing sensitivities. The color of the sensitivity bars indicates the direction of the relationship between the variable and 10-year proportional change in within-herd prevalence (grey indicates the effect of variable values below their mean value, white indicates the effect of values above their mean, and black indicates that the effect is unclear).

**Figure 9:**
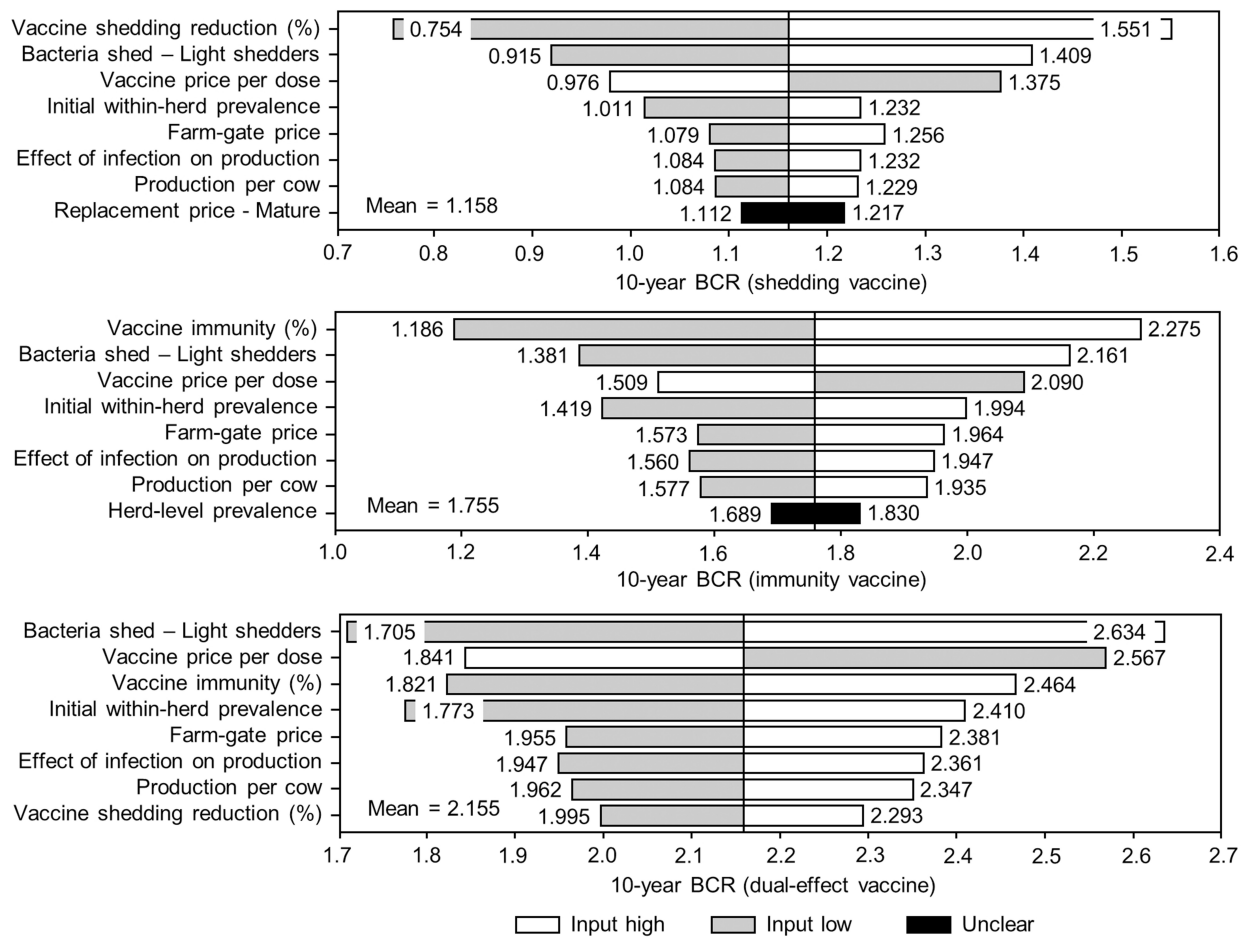
Sensitivity of 10-year benefit-cost ratios (BCRs) associated with various JD (paratuberculosis) vaccine types in average Canadian dairy herds to a range of input variables. Assumes initial mean values of 10% for within-herd *Mycobacterium avium* subsp. *paratuberculosis* (MAP) infection prevalence, 50% for herd-level prevalence, and 50% for vaccine efficacies. The color of the sensitivity bars indicates the direction of the relationship between the variable and 10-year BCR (grey indicates the effect of variable values below their mean value, white indicates the effect of values above their mean, and black indicates that the effect is unclear).

**Figure 10:**
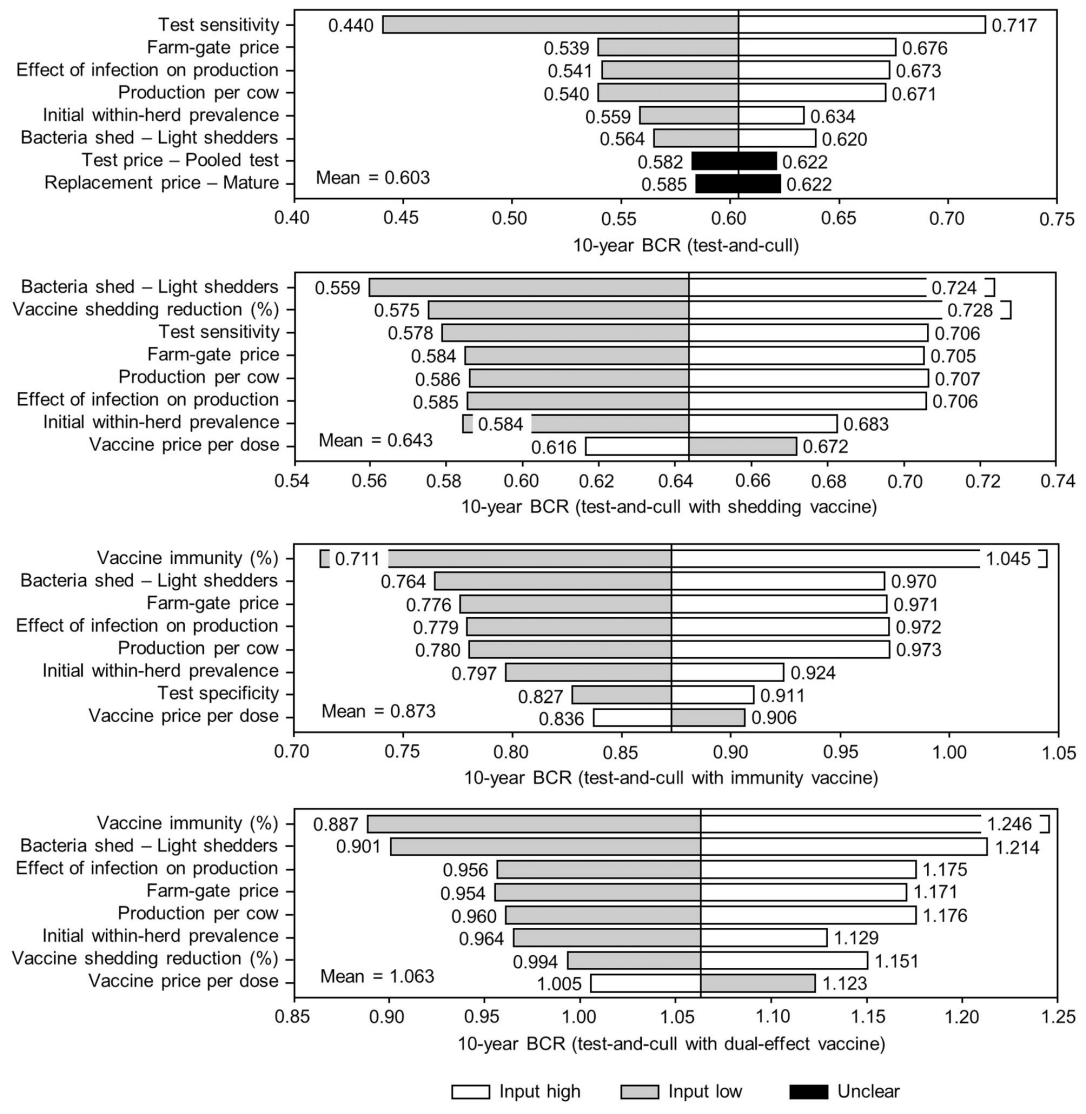
Sensitivity of 10-year benefit-cost ratios (BCRs) associated with various JD (paratuberculosis) control practices involving testing and culling in average Canadian dairy herds to a range of input variables. Assumes initial mean values of 10% for within-herd *Mycobacterium avium* subsp. *paratuberculosis* (MAP) infection prevalence, 50% for herd-level prevalence, 50% for vaccine efficacies, and 50% for testing sensitivities. The color of the sensitivity bars indicates the direction of the relationship between the variable and 10-year BCR (grey indicates the effect of variable values below their mean value, white indicates the effect of values above their mean, and black indicates that the effect is unclear).

The sensitivity of the proportional changes in within-herd prevalence over the 10-year horizons to a variety of input variables based on 10,000 iteration Monte Carlo simulations are presented in Figures 7, 8. In the shedding reduction vaccine scenario, the proportional change was most sensitive to the initial within-herd prevalence, with above-mean within-herd prevalence values resulting in lesser proportional increases and therefore more effective JD control. Other impactful and negatively related variables were the shedding reduction efficacy of the vaccine and the additional culling risk associated with Stage 1 MAP infection. The degree of bacterial shedding among lightly shedding infected animals and herd-level prevalence were also determined to be impactful, but positively related to the proportional increase in within-herd prevalence, with above-mean values resulting in greater proportional increases in within-herd prevalence. The protective immunity vaccine estimate was sensitive to similar variables, with the percentage of protective immunity being the most impactful, as was the dual-effect vaccine scenario estimate, with protective immunity having a significantly larger impact than shedding reduction. In all scenarios involving testing and culling, both alone and in combination with some type of vaccination, proportional changes to within-herd prevalence were most sensitive to test sensitivity, with initial within-herd prevalence, vaccine efficacy, and the degree of bacterial shedding among lightly shedding animals being consistently impactful to lesser degrees. Similar variables were identified as impactful in the 10,000 iteration Monte Carlo simulation sensitivity analyses of estimated 10-year BCRs using an average Canadian dairy herd (Figures 9, 10).

The stochasticity introduced through the Monte Carlo simulations resulted in values ranging from ~5 to 15% for the initial within-herd prevalence over the 10,000 iterations, with the 10-year proportional change in within-herd prevalence varying accordingly, as presented in Figure 7 through Figure 10. However, additional economic and production variables such as the vaccine price per dose, farm-gate price of milk, annual production per cow, and the effect of MAP infection on milk production were also identified. The degree of bacterial shedding among lightly shedding animals was once again consistently found to be impactful and positively related to BCR estimates in all scenarios. All significantly impactful variables in these BCR sensitivity analyses were positively related to estimated BCRs, aside from the vaccine price per dose, which was negatively related. In all control scenarios, within-herd prevalence was inversely related to the 10-year proportional change in within-herd prevalence and directly related to the benefit-cost ratio of the control practice.

## Discussion

With the assumptions of mean within-herd MAP infection prevalence of 10%, a mean herd-level MAP infection prevalence of 50%, vaccine efficacies (reduction in shedding and protective immunity) of 50%, mean test sensitivity of 50%, and mean test specificity of 99%, no scenarios resulted in the elimination of JD within a 10-year horizon. However, all control practices reduced within-herd MAP prevalence relative to no intervention within a 10-year horizon. However, at the 50% vaccine efficacy and 50% test sensitivity level, the only control practices that resulted in absolute reductions relative to initial within-herd MAP prevalence within the horizon were dual-effect vaccines, and protective immunity and dual-effect vaccines combined with testing and culling. Testing and culling alone did not; after three to four periods, an upward trend in within-herd prevalence was observed as new MAP infections occurred. Kudahl et al. (31) found that testing and culling alone only delayed an increase in within-herd prevalence, whereas Kirkeby et al. (20) found that that even with currently available testing tools, eradication of JD was attainable within seven to 10 years through testing and culling in Danish dairy herds. However, in the latter model, MAP infection was treated as an endemic situation, and therefore modeled using a density-dependent transition model as opposed to modeling the probability of infection as a function of the number and degree of infected animals in the herd. Also, their model explicitly considered a range of hygiene levels across herds, whereas in this model, variations in herd hygiene are instead implicitly captured using a range of possible disease progression rates and MAP-specific input variables. The upward trend observed in the testing and culling scenarios was also accentuated by the 10-year horizon of the simulations; at test sensitivity levels in the 50–70% range, testing, and culling did not lower infection pressure within the herd quickly enough to overcome the disease progression of false-negative, subclinically infected, and non-shedding animals to stages of infection characterized by moderate and heavy shedding. As infections in those strata progressed, infection pressure within the herd, and therefore within-herd prevalence, began to rise again. If testing and culling were continued, with each passing 5- or 10-year horizon these oscillations would lessen in amplitude and an overall downward trend would be observed. However, from an economic and epidemiologic modeling perspective, it is unrealistic to assume that herd compositions, management techniques, testing procedures, and even market structures would remain unchanged for more than 10 years. Therefore, the time horizon of the model was not extended.

Control variable values such as vaccine efficacy and testing sensitivity were clearly impactful on the effectiveness (ability to reduce within-herd prevalence within a 10-year period), economic impact (the ratio of benefits to costs per cow accrued as a result of implementation), and break-even period (years for cumulative benefits to equal cumulative costs). The results suggest that the effectiveness of the dual-effect vaccine was primarily driven by the protective immunity effect of the vaccine as opposed to the shedding reduction effect. At higher ranges of protective immunity, the reduced-shedding effect of the dual-effect vaccine ceased to have impact on the final MAP prevalence; at levels >80% protective immunity, reduced shedding among MAP-positive animals actually had the reverse effect, resulting in a final prevalence greater than the final prevalence that would have been achieved using a single-effect protective immunity vaccine. In the model, disease progression is related to the degree and number of shedding animals in the herd. Therefore, a reduction in shedding among MAP-infected animals resulted in less severe but more prolonged subclinical infections; these non-shedding, subclinically infected animals remained in the herd rather than developing clinical signs of JD and being culled. Once again, if the horizon of the model were extended by five or 10 periods, this result would likely not be observed as the remaining subclinically infected animals would eventually exit the herd. However, for reasons already described, the model was not extended past its 10-year horizon.

Through the Monte Carlo sensitivity analyses, the degree of bacterial shedding among lightly shedding animals was identified as an impactful variable, highlighting the need for further research into this area. Also impactful were the farm-gate price of milk and annual production per cow due to their positive relationships with production, and therefore forgone production losses due to MAP infection. For the selection of major dairy-producing regions that were modeled, production benefits were measured as potential increases in milk sales. Dual-effect vaccines were among the most successful control practices in terms of their reduction in within-herd prevalence and were economically viable with BCRs greater than one in all countries except Poland, Brazil, China, Russia, and Turkey. These countries are five of the seven countries with the lowest annual milk production per cow that were modeled, along with Ireland and New Zealand. However, Ireland and New Zealand have significantly greater aggregated salvage prices and replacement costs than the other five countries. The combination of relatively low costs and low annual production resulted in lower economic losses due to JD, and therefore less economic benefits from controlling JD in those five countries.

Two interesting patterns emerged across a range of control variable values (test sensitivity, shedding reduction, and protective immunity), both related to testing and culling. Firstly, testing and culling and testing and culling combined with a protective immunity vaccine were the only control scenarios where estimated annual costs per cow increased as the control variable values increased. In the vaccine scenarios without testing and culling, as within-herd MAP prevalence decreased with more effective controls, the culling rate also decreased as overall herd health improved. Because the vaccine was only administered to natural and purchased replacements after the initial time 0 whole-herd vaccination, costs per cow decreased over time as there were relatively fewer replacements requiring vaccination in each period. However, with testing and culling, this effect was outweighed by the fact that a more sensitive test detected more positive animals, which then needed to be culled and replaced at a relatively high cost. While testing and culling was effective at reducing within-herd prevalence relative to its initial value at test sensitivities >70%, this effectiveness depended entirely on aggressive culling of test-positive animals which may be impractical in a real-world setting, particularly in moderate and high prevalence herds. Similarly, in their simulations, Groenendaal et al. (32) found that while a test with 80% sensitivity in all infected animals was effective at reducing within-herd prevalence, the strategy was economically unviable because of the high culling rate of test-positive animals, particularly young ones, required to achieve that reduction in prevalence. Unless the costs of replacing test-positive and subsequently culled animals can be reduced for producers, this model also suggests that the benefits of testing and culling may not equal or exceed the costs, even if new, more sensitive and specific tests are developed. However, it is important to note that the simulated testing protocol remained static throughout the 10-year horizon; a desirable real-world testing and culling program would not only need to reduce replacement costs, but also reduce testing costs by using a dynamic testing strategy (e.g., environmental testing instead of pooled and individual testing once within-herd prevalence is reduced to a certain level). For herds with low initial within-herd prevalence, a dynamic testing strategy alone could reduce costs to the point where testing and culling becomes economically viable, particularly in closed herd scenarios where all replacements come from within the herd. If more sensitive tests were also developed, these low prevalence closed herds could become reliable and certifiable sources of MAP-negative replacements for higher prevalence open herds seeking to reduce within-herd MAP prevalence or low prevalence herds seeking to rapidly expand, with these replacements potentially being sold at an economic premium. The second interesting pattern that emerged related to testing and culling was that when combined with a vaccine that reduced shedding and when combined with a dual-effect vaccine, benefits per cow decreased as the control variable values (vaccine efficacy and test sensitivity) increased from 70 to 90%. Because a fecal PCR test was modeled, the test could only detect animals in shedding states of infection. Therefore, as the shedding-reducing effects of the vaccine were increased, the number of animals detectable by fecal PCR testing was reduced, and the prevalence-reducing effects of improved testing sensitivity were partially offset. Because of this reduced ability to detect positive animals, the replacement costs associated with testing and culling also decreased. When these decreased costs were combined with the overall improvement in herd health due to vaccination and less aggressive testing and culling, the total costs per cow decreased at a greater rate than did benefits; the BCRs still increased with the control variable values despite the combination of vaccine-induced shedding reduction and fecal PCR testing being relatively inefficient.

While the general method described is appropriate for most dairy industries, the Canadian industry requires special attention. Canada's dairy sector operates with planned and controlled production levels, administered cost-of-production-based pricing, and import controls. There are two consequences relevant to this model: (i) production losses, a significant contributor to the benefits of JD control, can no longer be measured as forgone milk sales due to the production quota system; and (ii) Canada's above-average farm-gate price, which is the highest among countries modeled (aside from Japan) and much higher than the farm-gate price in the United States, Canada's most comparable counterpart. Apart from a higher level of annual output in the United States, both countries have similar dairy sector characteristics in terms of genetics, marketing, consumer preferences, and annual production per cow, and assuming the same within-herd and herd-level MAP prevalence across the two countries, there should be similar per-cow benefits and costs associated with controlling JD. However, the above average farm-gate price in Canada results in a greater valuation of production losses and therefore benefits from JD control in Canada. While these differences are attributable in part to differing technical and allocative efficiencies across US and Canadian dairy sectors, which are not addressed by this study, the effects of the differing market structures are addressed; to reflect the constraint of fixed production, production losses were also estimated as the cost of having additional, less productive MAP-positive cows to maintain a fixed level of production. Once adjusted, the estimated BCRs of all control practices in Canada dropped and their break-even periods increased. For example, the Canadian revenue-weighted average BCR for dual-effect vaccination at 50% efficacy decreased from 2.13 to 1.48 when production levels were treated as fixed. While this is more in line with the BCR of 1.66 in average US herds for the same type of vaccination, this may be an overcorrection. Although overall production and farm-gate prices in Canada are predetermined and producers are not paid for production that exceeds their quota-based targets, the overall level of production generally increases year-over-year (33) and producers trade quota through an exchange market; essentially, more technically efficient producers purchase quota from less technically efficient ones to increase the size of their operations. Evidence of this competition is clear: the number of dairy farms in Canada has steadily decreased over the last several decades while the size of herds has increased (34). In other words, Canadian producers operate in an environment between fixed production and pure competition. Therefore, the true BCRs of the various potential JD control practices for Canadian dairy herds likely lie between the fixed production and variable production estimates.

Finally, it is also important to recognize the limitations of this study. The net costs associated with a higher culling rate may be overestimated in this model. Because only the economic impacts of culling due to MAP-infection were considered, this model ignores the potential benefits associated with having a greater proportion of younger animals in the herd. For example, age-related conditions such as reduced fertility, mastitis, and lameness are all potential sources of economic losses that could be partially offset as a direct result of an increased cow-culling rate. Also, the production benefits due to an increased conception rate resulting from JD control were not explicitly estimated. Instead, these benefits were only implicitly considered through the variations around the mean milk yield reduction estimated by McAloon et al. (8). Lastly, it is also important to note that production systems, grazing periods, cattle breeds, etc. were assumed to be uniform across herds within regions at the mean level. However, variations in these production factors were implicitly captured through variations around the mean values used in the 10,000 iteration simulations.

## Conclusions

Vaccination was the most economically viable type of JD control practice modeled, with dual-effect vaccines (reducing shedding and providing protective immunity) being the most promising. Even with modest 50% reductions in shedding and 50% protective immunity conferred by vaccination, BCRs for this type of vaccine were between 2.13 and 1.48 in Canada, with a break-even period of between 6.17 and 7.61 years. At this same level of efficacy, dual-effect vaccines were also estimated to be desirable with BCRs greater than one in almost all major-dairy producing regions, with a revenue-weighted average BCR of 1.24 and a revenue-weighted average break-even period of 7.88 years. Testing and culling was comparably effective to a dual-effect vaccine at test sensitivities >70% but would remain economically unviable in almost all regions modeled, even at levels of testing sensitivity above 70%. The results suggest that the main barrier to testing and culling programs for JD is the impractical nature of the aggressive culling that would have to accompany highly sensitive tests. Without a reduction in the replacement cost of culled animals, vaccination, particularly dual-effect vaccination, is the most promising potential JD control practice for dairy producers. This research is an important contribution to the policy discussion surrounding paratuberculosis control in Canada and internationally.

## Data Availability Statement

The raw data supporting the conclusions of this article will be made available by the authors, without undue reservation.

## Author Contributions

PR and DH conceived of the research and developed the models. PR performed the simulations and computations. HB and DH verified the methodology and validity of the results. HB provided expertise and knowledge regarding MAP transmission and existing control practices. DH supervised this research. All authors discussed the results and reviewed the final manuscript.

## Conflict of Interest

The authors declare that the research was conducted in the absence of any commercial or financial relationships that could be construed as a potential conflict of interest.
