# Corrigendum: Effectiveness and Economic Viability of Johne's Disease (Paratuberculosis) Control Practices in Dairy Herds

**DOI:** 10.3389/fvets.2021.657453

**Published:** 2021-02-22

**Authors:** Philip Rasmussen, Herman W. Barkema, David C. Hall

**Affiliations:** ^1^Department of Ecosystem and Public Health, University of Calgary, Calgary, AB, Canada; ^2^Department of Production Animal Health, University of Calgary, Calgary, AB, Canada

**Keywords:** MAP, Johne's disease, paratuberculosis, vaccination, testing and culling, control practice, Markov chain, economic analysis

In the original article, there was a typographical error. A correction has been made to the exponent on the final bracket of Materials and Methods, Testing and Culling, Paragraph 1, **Equation (1)**. The corrected paragraph appears below.

“In this control scenario, animals aged 1–7 years are tested annually using a combination of pooled and individual fecal PCR tests. They are first tested at time zero, and then retested after each transition period (year) along with purchased replacements aged 1–3 years, which are tested only at the individual level. For all testing periods, the probability of a pooled test containing samples from an *r* number of MAP-positive animals given the pool size *n*, or *pr*(*TP*) | *C*(*n, r*), is determined using the following equation:

(1) $$\begin{array}{r} pr\left(TP\right)\;|\;C\left(n,r\right)={\frac{n!}{r!*\left(n-r\right)!}}^{*}{\left(\frac{TP_{s}}{animals_{\left(1-7\right)}}\right)}^{r}*{\left(1-\left(\frac{TP_{s}}{animals_{\left(1-7\right)}}\right)\right)}^{n-r} \end{array}$$

where: *TP*_*s*_ equals the number of true positive animals aged 1–7 years in a shedding state and *animals*_(1−7)_ equals the number of animals aged 1–7 years in the herd. A testing pool size of five animals is assumed, or *n* = 5. Pooled tests and individual tests are assumed to share the same sensitivities and specificities, or that *se*_*p*_ = *se*_*i*_ and *sp*_*p*_ = *sp*_*i*_.”

The authors apologize for this error and state that this does not change the scientific results or conclusions of the article in any way. The original article has been updated.

