# Supplementary material for: Effectiveness and Economic Viability of Johne's Disease (Paratuberculosis) Control Practices in Dairy Herds
Source: Front Vet Sci. 2021 Jan 15;7:614727. doi: 10.3389/fvets.2020.614727 (PMC7843527; doi:10.3389/fvets.2020.614727)
Supplement: Supplementary file 1 [file Data_Sheet_1.pdf]

Supplementary Files

Effectiveness and economic viability of Johne's disease (paratuberculosis) control practices in dairy herds

Philip Rasmussen, [1] ***Herman W. Barkema,*** [2] ***David C. Hall*** [1*]

[1] Department of Ecosystem and Public Health, University of Calgary, Calgary, AB, Canada, [2] Department of Production Animal Health, University of Calgary, Calgary, AB, Canada

Table S1[1] | General input variables used in Markov chain simulations of the MAP (*Mycobacterium avium* subsp. *paratuberculosis*) -positive and control practice herd models.

Variable	Mean value	Unit	Source
Effect of infection on production	5.90	%	(1)
Replacement pool	5000.00	cows	Assumed
Replacement cost (labor)	2.00	hours	Assumed
Bacteria shed – Light shedders[1]	5.00	CFU	(2,3)
Bacteria shed – Moderate shedders[1]	25.00	CFU	(2,3)
Bacteria shed – Heavy shedders[1]	50.00	CFU	(2,3)
Weight at 0 to 3 months	74.24	kg	(4)
Weight at 3 to 6 months	141.82	kg	(4)
Weight at 6 to 9 months	214.70	kg	(4)
Weight at 9 to 12 months	286.52	kg	(4)
Weight at 12 to 15 months	354.86	kg	(4)
Weight at 15 to 18 months	425.32	kg	(4)
Weight at 18 to 21 months	477.63	kg	(4)
Weight at 21 to 24 months	524.05	kg	(4)
Weight at maturity (2 to 9 years)	680.39	kg	(4)
Value reduction – Stage 4 animals	0.31	proportion	(5)
Value reduction – Stage 3 animals[2]	0.29	proportion	Calculated
Value reduction – Stage 2 animals[2]	0.26	proportion	Calculated
Value reduction – Stage 1 animals[2]	0.15	proportion	Calculated
Culling risk – Stage 4 animals	3.20	ratio	(6)
Culling risk – Stage 3 animals[3]	2.98	ratio	Calculated
Culling risk – Stage 2 animals[3]	2.69	ratio	Calculated
Culling risk – Stage 1 animals[3]	1.08	ratio	Calculated

[1] Light and moderate shedding values based on median CFU count for the range. Heavy shedders at minimum cutoff.
[2] Based on Stage 4 value reduction observed in study. Other values estimated by scaling the Stage 4 value to a truncated cumulative logistic probability distribution (max=0.308, alpha=0.308, beta=0.031).
[3] Based on Stage 4 hazard ratio observed in the study. Other values estimated by scaling the Stage 4 value to a truncated cumulative logistic probability distribution (max=3.200, alpha=3.200, beta=0.320). Stage 1 risk is based on a mean value of 1.00 with a normal distribution and standard deviation of 0.10, truncated with a minimum value of 1.00 to obtain a true mean of 1.08.

[1] Tables S1, S2, and S3 taken from Working Paper *Economic losses due to JD in dairy herds* by Philip Rasmussen, Herman Barkema, Steve Mason, Eugene Beaulieu, and David Hall (2020) - University of Calgary, Dept. Ecosystem and Public Health, Faculty of Veterinary Medicine.

Table S2 | Region-specific dairy sector characteristics for major dairy-producing regions in order of decreasing 2018 annual production used in Markov chain simulations .

Region	Annual production ('000 MT)[1]	Annual production (kg/cow)[2]	Dairy cattle ('000 head)[3]	Farm-gate price (USD/100kg)[4]
European Union (28)	166,744	7,279	22,906	40.22
Germany	33,087	8,068	4,101	40.53
France	25,055	7,058	3,550	39.92
United Kingdom	15,488	8,243	1,879	39.07
Poland	14,171	6,401	2,214	37.71
Netherlands	14,090	9,079	1,552	42.50
Italy	12,340	7,289	1,693	44.08
Ireland	7,831	5,720	1,369	39.20
Spain	7,336	8,968	818	36.83
Denmark	5,615	9,851	570	42.41
Belgium	4,178	7,898	529	37.96
Austria	3,821	7,169	533	38.77
Czechia	3,162	8,808	359	39.33
Sweden	2,760	8,818	313	40.85
Finland	2,398	9,083	264	44.72
United States	98,688	10,546	9,358	35.86
California	18,331	10,572	1,734	35.86
Wisconsin	13,870	10,887	1,274	35.86
Idaho	6,871	11,283	609	35.86
New York	6,750	10,835	623	35.86
Texas	5,830	10,856	537	35.86
Michigan	5,066	11,947	424	35.86
Pennsylvania	4,838	9,321	519	35.86
Minnesota	4,476	9,881	453	35.86
New Mexico	3,758	11,388	330	35.86
Washington	3,055	11,030	277	35.86
Brazil[5]	33,491	1,963	17,060	41.71
China[5]	30,640	2,563	11,955	53.87
Russia	30,611	4,492	6,815	36.58
New Zealand	21,947	4,437	4,946	35.72
Turkey	20,037	3,161	6,338	40.22
Canada	10,228	10,519	972	53.93
Québec	3,673	10,369	354	53.11
Ontario	3,377	10,432	324	52.72
British Columbia	906	10,803	84	58.81
Alberta	867	10,965	79	55.95
Manitoba	448	10,880	41	54.14
Saskatchewan	329	11,256	29	53.74
Nova Scotia	226	10,566	21	53.17
New Brunswick	191	9,985	19	53.59
Prince Edward Island	151	10,494	14	54.02
Newfoundland and Labrador	64	10,778	6	70.74
Australia	9,176	6,017	1,525	34.00
Japan	7,290	8,607	847	93.15

[1] Canadian production values: CDIC - Average Production based on Official - Supervised Records (7). US annual production values: USDA ERS – Dairy data – Milk cows and production by State and region (8). All other regions: CLAL.it – Dairy by Country (9).
[2] Canadian annual per cow production values: CDIC - Average Production based on Official - Supervised Records (7). US production per cow values: USDA ERS – Milk cows and production by State and region (USDA ERS, 2019). All other regions: CLAL.it – Dairy by Country (9).
[3] Canadian cattle values: STATCAN – Table 32-10-0130-01 – Number of cattle, by class and farm type (x 1,000) (10). US cattle values: USDA ERS – Dairy data – Milk cows and production by State and region (8). All other regions: CLAL.it – Dairy by Country (9).
[4] Turkey: 2018 EU-28. Australian price: Australian Dairy Industry in Focus – 2018 (11). Canadian values: CDIC - MI011 - Canadian farm cash receipts from dairying (12). All other regions: CLAL.it – Dairy by Country (9). Converted to 2018 US$ using IRS.gov – Yearly Average Currency Exchange Rates (13).
[5] 2017 values.

Table S3 | Region-specific economic variables used in the Monte Carlo simulations of the MAP (*Mycobacterium avium* subsp. *paratuberculosis*) -positive Markov herd model. All variables simulated with a normal distribution and standard deviation of 10% of the mean.

Region	gdppc[1] (USD)	wage[2] (USD /hr)	r-c[3] (USD/ hd)	r-oh[3] (USD /hd)	r-bh[3] (USD /hd)	r-m[3] (USD /hd)	s-12[4] (USD /kg)	s-24[4] (USD /kg)	s-m[4] (USD /kg)
European Union	39,928	10.08	59.94	375.71	650.80	764.17	2.12	1.69	0.71
Germany	47,603	12.11	72.01	451.35	781.82	918.02	2.55	2.03	0.85
France	41,464	10.39	61.78	387.19	670.69	787.52	2.19	1.74	0.73
United Kingdom	42,944	10.53	62.62	392.48	679.86	798.29	2.22	1.76	0.74
Poland	15,421	3.65	21.71	136.05	235.66	276.71	0.77	0.61	0.26
Netherlands	53,024	14.15	84.11	527.18	913.17	1,072.25	2.98	2.37	0.99
Italy	34,483	9.54	56.73	355.59	615.94	723.24	2.01	1.60	0.67
Ireland	78,806	19.39	115.29	722.64	1,251.75	1,469.81	4.08	3.25	1.36
Spain	30,371	7.02	41.75	261.65	453.24	532.19	1.48	1.18	0.49
Denmark	61,350	16.33	97.10	608.60	1,054.22	1,237.87	3.44	2.73	1.15
Belgium	47,519	11.32	67.32	421.97	730.94	858.27	2.39	1.90	0.79
Austria	51,462	12.53	74.47	466.79	808.56	949.41	2.64	2.10	0.88
Czechia	23,079	5.70	33.88	212.33	367.79	431.86	1.20	0.95	0.40
Sweden	54,608	14.00	83.26	521.84	903.92	1,061.39	2.95	2.34	0.98
Finland	50,152	14.08	83.70	524.64	908.77	1,067.08	2.97	2.36	0.99
United States	62,795	14.14	84.05	526.79	912.50	1,071.46	2.98	2.37	0.99
California	60,359	13.59	80.79	506.36	877.11	1,029.90	2.86	2.27	0.95
Wisconsin	48,666	10.96	65.14	408.26	707.19	830.38	2.31	1.83	0.77
Idaho	36,441	8.20	48.77	305.71	529.54	621.79	1.73	1.37	0.58
New York	65,220	14.68	87.29	547.14	947.74	1,112.84	3.09	2.46	1.03
Texas	53,737	12.10	71.92	450.80	780.88	916.91	2.55	2.02	0.85
Michigan	44,201	9.95	59.16	370.81	642.31	754.20	2.10	1.67	0.70
Pennsylvania	51,841	11.67	69.39	434.90	753.33	884.56	2.46	1.95	0.82
Minnesota	54,805	12.34	73.35	459.76	796.40	935.13	2.60	2.06	0.87
New Mexico	41,619	9.37	55.71	349.15	604.79	710.14	1.97	1.57	0.66
Washington	59,333	13.36	79.41	497.75	862.20	1,012.39	2.81	2.24	0.94
Brazil	8,921	2.34	13.89	87.04	150.78	177.04	0.49	0.39	0.16
China	9,771	3.30	19.64	123.13	213.28	250.43	0.70	0.55	0.23
Russia	11,289	2.59	15.41	96.60	167.34	196.49	0.55	0.43	0.18
New Zealand	41,945	9.41	55.92	350.49	607.12	712.89	1.98	1.57	0.66
Turkey	9,370	2.37	14.07	88.17	152.73	179.34	0.50	0.40	0.17
Canada	46,269	15.66	93.13	583.69	1,011.06	1,187.19	3.30	2.62	1.10
Québec	40,389	13.47	80.06	501.77	869.16	1,020.57	2.84	2.25	0.95
Ontario	46,167	15.28	90.85	569.43	986.36	1,158.18	3.22	2.56	1.07
British Columbia	45,540	16.81	99.96	626.55	1,085.31	1,274.38	3.54	2.81	1.18
Alberta	61,816	21.71	129.08	809.03	1,401.40	1,645.53	4.57	3.63	1.52
Manitoba	41,409	14.08	83.68	524.51	908.55	1,066.82	2.96	2.36	0.99
Saskatchewan	53,487	18.04	107.27	672.37	1,164.68	1,367.57	3.80	3.02	1.27
Nova Scotia	35,641	11.90	70.73	443.34	767.95	901.73	2.51	1.99	0.84
New Brunswick	36,970	12.44	73.94	463.44	802.76	942.60	2.62	2.08	0.87
Prince Edward Island	35,111	11.91	70.79	443.67	768.53	902.41	2.51	1.99	0.84
Newfoundland and Labrador	48,761	21.57	128.25	803.86	1,392.44	1,635.01	4.54	3.61	1.51
Australia	57,374	12.25	72.81	456.34	790.46	928.16	2.58	2.05	0.86
Japan	39,290	22.98	136.60	856.16	1,483.02	1,741.37	4.84	3.84	1.61

[1] Gross domestic product per capita (gdppc). US GDPPC by state (14) and Canadian GDP by province (15). Converted to US$ (13). All other regions (16).

[2] Estimated aggregate dairy wage rate (wage). US 2018 (17). All other regions (*i* through *n*) calculated using the following formula: $wage_i = \frac{wage_{USA} * gdppc_i * farm-gate\ price_i}{farm-gate\ price_{USA} * gdppc_{USA}}$.

[3] Calf ("c"), open heifer ("oh"), bred heifer ("bh") and mature cow ("m") replacement ("r") costs. US replacement prices (18). All other regions (*i* through *n*) calculated using the following formula: $r_i = \frac{r_{USA} * gdppc_i * farm-gate\ price_i}{farm-gate\ price_{USA} * gdppc_{USA}}$.

[4] 0-12-month animals ("12"), 12-24-month animals ("24"), and mature cow ("m") salvage ("s") prices. Canadian salvage prices (19). Converted to kg at 50.8023 kg/CWT and converted to US$ (13). All other regions (*i* through *n*) calculated using the following formula: $s_i = \frac{s_{CAN} * gdppc_i * farm-gate\ price_i}{fram-gate\ price_{CAN} * gdppc_{CAN}}$.

References – Supplementary Files

1. McAloon CG, Whyte P, More SJ, Green MJ, O'Grady L, Garcia A, Doherty ML. The effect of paratuberculosis on milk yield—A systematic review and meta-analysis. *J. Dairy Sci.* (2016) 99: 1449-1460. doi:10.3168/jds.2015-10156
2. Crossley BM, Zagmutt-Vergara FJ, Fyock TL, Whitlock RH, Gardner IA. Fecal shedding of *Mycobacterium avium* subsp. *paratuberculosis* by dairy cows. *Vet. Microbiol.* (2005) 107: 257-263. doi:10.1016/j.vetmic.2005.01.017
3. Whitlock RH, Wells SJ, Sweeney RW, Van Tiem J. ELISA and fecal culture for paratuberculosis (Johne's disease): sensitivity and specificity of each method. *Vet. Microbiol.* (2000) 77: 387-398. doi:10.1016/S0378-1135(00)00324-2
4. Jones CM, Heinrichs J. Growth Charts for Dairy Heifers: Comparing to a breed standard can indicate if heifer growth is progressing normally. *Penn State Extension* (2017) Available at: https://extension.psu.edu/growth-charts-for-dairy-heifers [Accessed January 17, 2020].
5. Kudahl AB, Nielsen SS. Effect of paratuberculosis on slaughter weight and slaughter value of dairy cows. *J. Dairy Sci.* (2009) 92: 4340-4346. doi:10.3168/jds.2009-2039
6. Hendrick SH, Kelton DF, Leslie KE, Lissemore KD, Archambault M, Duffield TF. Effect of paratuberculosis on culling, milk production, and milk quality in dairy herds. *J. Am. Vet. Med. Assoc.* (2005) 227: 1302-1308. doi:10.2460/javma.2005.227.1302
7. CDIC. D037-3 - Average Production by Province. *Canadian Dairy Information Centre* (2019) Available at: https://aimis-simia-cdic-ccil.agr.gc.ca/rp/index-eng.cfm?action=pR&r=215&pdctc= [Accessed October 10, 2019].
8. USDA ERS. Dairy data – milk and cow production by State and region. *United States Department of Agriculture - Economic Research Service* (2019) Available at: https://www.ers.usda.gov/webdocs/DataFiles/48685/milkcowsandprod_1_.xlsx?v=2695 [Accessed January 14, 2020].
9. CLAL. Dairy Sector by Country. *CLAL S. R. L.* (2019) Available at: https://www.clal.it/en/ [Accessed September 15, 2020].
10. STATCAN. Table 32-10-0130-01 Number of cattle, by class and farm type (x 1,000). *Statistics Canada* (2020) Available at: https://www150.statcan.gc.ca/t1/tbl1/en/tv.action?pid=3210013001 [Accessed September 13, 2020].
11. Dairy Australia. Dairy in Focus - 2018. *Dairy Australia* (2019) Available at: https://www.dairyaustralia.com.au/publications/australian-dairy-industry-in-focus-2018?id=B81A5CE26AAE4C0F898E8F3BFF0014D9 [Accessed January 26, 2020].
12. CDIC. MI011 - Canadian farm cash receipts from dairying. *Canadian Dairy Information Centre* (2019) Available at: https://aimis-simia-cdic-ccil.agr.gc.ca/rp/index-eng.cfm?action=pR&r=272&pdctc= [Accessed September 14, 2020].
13. IRS. Yearly average currency exchange rates. *Internal Revenue Service* (2020) Available at: https://www.irs.gov/individuals/international-taxpayers/yearly-average-currency-exchange-rates [Accessed January 13, 2020].
14. BEA. Regional Economic Accounts – SAGDP Tables: Annual GDP by State. *Bureau of Economic Analysis* (2019) Available at: https://www.bea.gov/data/gdp/gdp-state [Accessed January 13, 2020].
15. STATCAN. Table 36-10-0222-01 Gross domestic product, expenditure-based, provincial and territorial, annual (x 1,000,000). *Statistics Canada* (2019) Available at: https://www150.statcan.gc.ca/t1/tbl1/en/tv.action?pid=3610022201 [Accessed January 14, 2020].

16. World Bank. GDP per capita (current US$). *World Bank* (2020) Available at: https://data.worldbank.org/indicator/NY.GDP.PCAP.CD [Accessed January 4, 2020].
17. USDA NASS. Farm Labor – 05/30/2019. *United States Department of Agriculture, National Agricultural Statistics Service* (2019) Available at: https://www.nass.usda.gov/Publications/Todays_Reports/reports/fmla0519.pdf [Accessed January 19, 2020].
18. USDA. National Dairy Comprehensive Report - Monthly - 02/29/2020. *United States Department of Agriculture* (2020) Available at: https://www.ams.usda.gov/mnreports/lsmdairycomp.pdf [Accessed February 11, 2020].
19. STATCAN. Table 32-10-0077-01 Farm product prices, crops and livestock. *Statistics Canada* (2019) Available at: https://www150.statcan.gc.ca/t1/tbl1/en/tv.action?pid=3210007701 [Accessed January 20, 2020].
